# Translation and validation of Indonesian version of Pediatric Quality of Life Inventory™ (PedsQL™) Neuromuscular Module

**DOI:** 10.1186/s12955-022-01933-x

**Published:** 2022-02-24

**Authors:** Guswan Wiwaha, Dian M. Sari, Vitriana Biben, Deni K. Sunjaya, Dany Hilmanto

**Affiliations:** 1 Department of Public Health, Faculty of Medicine Universitas Padjadjaran/Dr. Hasan Sadikin General Hospital, Eyckman 38, Bandung, West Java 40161 Indonesia; 2 Department of Physical Medicine and Rehabilitation, Faculty of Medicine Universitas Padjadjaran/Dr. Hasan Sadikin General Hospital, Eyckman 38, Bandung, West Java 40161 Indonesia; 3 Department of Child Health, Faculty of Medicine Universitas Padjadjaran/Dr. Hasan Sadikin General Hospital, Eyckman 38, Bandung, West Java 40161 Indonesia

**Keywords:** Child, Indonesia, Neuromuscular diseases, Psychometric, Quality of life, Rasch model, The PedsQL™ 3.0 Neuromuscular Module Indonesian Version

## Abstract

**Background:**

The Pediatric Quality of Life™ 3.0 Neuromuscular Module is an instrument to assess health-related quality of life (HRQoL) among children with neuromuscular diseases (NMDs) aged 2–18 years. This study aimed to determine whether the PedsQL™ 3.0 Neuromuscular Module Indonesian Version is valid and reliable.

**Methods:**

This study used the Indonesian translation of the PedsQL™ 3.0 Neuromuscular Module after getting formal permission from the inventor, and the translation process followed the Mapi linguistic translation guidelines. This study administered the PedsQL™ 3.0 Neuromuscular Module Indonesian Version to 84 parents and 71 children. In addition, we used the Rasch model to analyze the psychometric properties.

**Results:**

The reliability of the total scale of the PedsQL™ Neuromuscular Module Indonesian Version shows good to very good criteria. On the parent proxy-report, Cronbach alpha was 0.95, person reliability was 0.84, item reliability was 0.93, person separation was 2.32, item separation was 3.61, person strata separation was 4 levels, and item strata separation was 5 levels. On the child self-report, Cronbach alpha was 0.93, person reliability was 0.81, item reliability was 0.81, person separation was 2.08, item separation was 2.06, person strata separation was 3 levels, and item strata separation was 3 levels. The total scale of the PedsQL Neuromuscular Module Indonesian Version shows fair to good construct validity in parent proxy-report (explained variance 51.9%; unexplained variance 8.4%) and child self-report (explained variance 40.9%; unexplained variance 12.6%). There were no misfit items in the parent proxy-report (infit 0.66–1.49; outfit 0.51–1.81; point measure correlation 0.36–0.93) and child self-report (infit 0.53–1.65; outfit 0.50–1.73; point measure correlation 0.31–0.90) identified by the Rasch models.

**Conclusions:**

The PedsQL™ 3.0 Neuromuscular Modul Indonesian Version is a valid and reliable instrument in measuring HRQoL in Indonesian children with neuromuscular diseases.

**Supplementary Information:**

The online version contains supplementary material available at 10.1186/s12955-022-01933-x.

## Background

Neuromuscular diseases (NMDs) are a heterogeneous group of diseases that affect skeletal muscles. Abnormalities in the anterior horn motor cells, peripheral nerves, neuromuscular junctions, or muscles can lead to inherited or acquired NMDs [1, 2]. Spinal Muscular Atrophy (SMA) and Duchenne Muscular Dystrophy (DMD) are the most common neuromuscular diseases that affect children [1, 3, 4].

NMDs affect 0.1% of the total UK population, with a prevalence of 37 per 100.000 [5]. Incidence of all types of SMA is 1 in 10.000 live births, the most cases with a prevalence of around 1–2 per 100.000 persons [6]. Even though the incidence was relatively low, patients with NMDs have some difficulties that often interfere their daily activities. The impairments of NMDs patients include swallowing, feeding, muscle weakness, joint contracture, and respiratory problems; thus, their quality of life decreases [7].

The quality of life of NMDs patients can be measured with the Health-Related Quality of Life questionnaire (HRQoL), a multidimensional instrument consisting of physical, psychological (including emotional and cognitive), and social health subscales. HRQoL has emerged as being the most appropriate term to measure the quality of life that represent a patient’s perceptions of the impact of an illness and its treatment on their functioning and well-being and which are within the scope of healthcare services and medical products to gain a comprehensive evaluation of the patients [8].

The Pediatric Quality of Life Inventory (PedsQL) is an instrument to assess HRQoL in children and adolescents aged 2 to 18. It consists of generic core and disease-specific modules [8]. The PedsQL™ 3.0 Neuromuscular Module was developed by Dr. James W. Varni and a disease-specific module for measuring children's quality of life with NMDs by assessing their neuromuscular disease, communication difficulties, and family resources. This instrument is acknowledged as a validated health outcome measure in patients with NMDs and demonstrated good reliability for the total score in SMA patients (Cronbach’s alpha = 0.89/parent proxy-report, 0.88/child self-report; intraclass correlation coefficients/ICC = 0.89/parent proxy-report, 0.81/child self-report) [8]. This instrument has also been translated and validated in China [9] for DMD patients, while in Thailand [10] for NMDs patients consisting of DMD, Charcot Marie Tooth (CMT), SMA, and others. Health professionals can use the PedsQL™ 3.0 Neuromuscular Module to review and evaluate the management and treatment of children with NMDs. However, this PedsQL™ 3.0 Neuromuscular Module officially is not yet available in the Indonesian version since it is not yet validated nor measured the reliability in Indonesia.

From previous studies, the PedsQL™ 3.0 Neuromuscular Module properties were underpinned by the Classical Test Theory (CTT) [8–10].The most common approach to measuring the psychometric quality of the instruments and rating scales is using principles of Classical Test Theory (CTT), which has several limitations. The CTT model has a raw score bias in favor of the central and against the extremes scores, implying that raw scores are always target-biased and sample-dependent.

The Rasch model can tackle many deficiencies of the CTT model, such as incorporating missing data, producing validity and reliability measures for person measures and item calibrations, measuring persons and items on the same metric, and being sample-free [11].

Thus, this study aimed to determine the PedsQL™ 3.0 Neuromuscular Module's validity and reliability in the Indonesian version of the PedsQL™ 3.0 Neuromuscular Module using the Rasch model.

## Methods

**Fig. 1:**
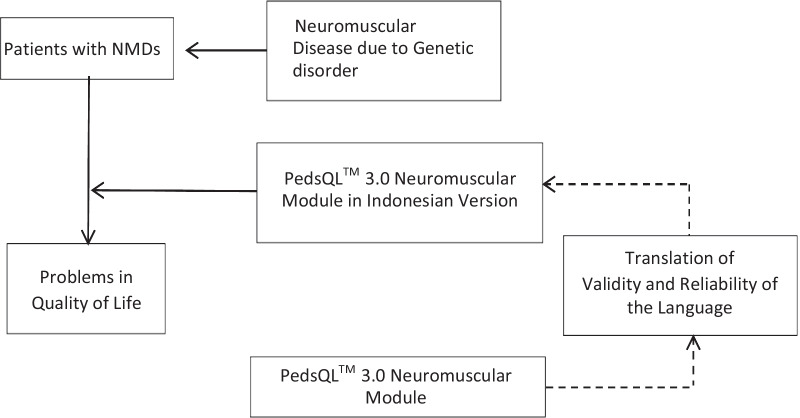
Diagram of the concept

This study used a mixed-methods with an exploratory sequential design. First, it began qualitatively to obtain a standard Indonesian version (PedsQL™ 3.0 Neuromuscular Module Indonesian Version) followed by a descriptive quantitative study to get the PedsQL™ 3.0 Neuromuscular Module Indonesian Version’s validity and reliability (Fig. 1).

The PedsQL™ 3.0 Neuromuscular Module was used as the object of the qualitative method. This module that has 25 items for total scales consists of three subscales: (1) About My/My Child’s Neuromuscular Disease subscale (17 items related to the disease process and associated symptomatology), (2) Communication subscale (3 items related to the patient’s ability to communicate with health care providers and others about his/her illness), and (3) About Our Family Resources subscale (5 items related to family financial and social support system). The module comprises parent proxy-report and child self-report formats for children. It is divided into seven age groups, including parent proxy-report formats for ages 2–4, 5–7, 8–12, and 13–18 years and child self-report formats for ages 5–7, 8–12, and 13–18 years. The young child form (ages 5–7 years) does not contain the “Communication” or “About Our Family Resources” subscales. The participants were asked how much of a problem each item had been during the past month. Responses are rated for child self-report for children, teens, and parent proxy-reports using a 5-point Likert scale (0 = never a problem, 1 = almost never a problem, 2 = sometimes a problem, 3 = often a problem, 4 = almost always a problem). The self-administered form used for the 5–7 years group uses a 3-point scale consisting of emojis to obtain the level of difficulty (0 with a big smiling emoji = not at all a problem; 2 with an indifferent emoji = sometimes a problem; and, 4 with a sad emoji = almost always a problem) [12].

The instrument was then translated forward and backward by licensed translators. The expert panel supervised and controlled the translation process to ensure the proper collection of information obtained from the instrument.

**Fig. 2:**
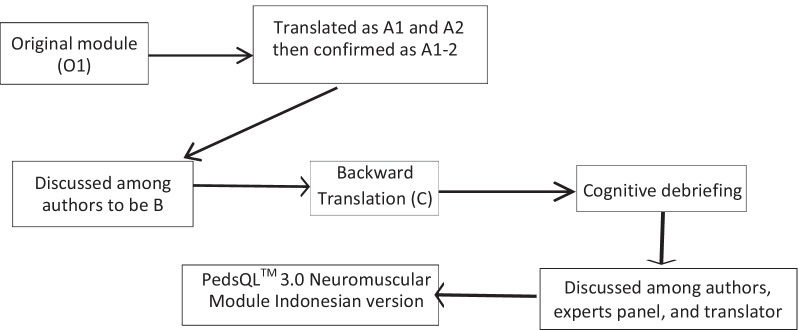
Diagram of the steps in this study

This study was conducted in 5 steps: the initial translation (forward translation), synthesis of combined translation, backward translation, cognitive debriefing, then validity and reliability trials of the PedsQL™ 3.0 Neuromuscular Module Indonesian Version (Fig. 2). All steps were done, and the final version was accepted on behalf of Dr. James W. Varni by MAPI Research Institute in Lyon, France.

For the cognitive debriefing on qualitative methods, we used parents and children with NMDs aged 2 to 18 years at the Medical Rehabilitation Polyclinic of Dr. Hasan Sadikin Hospital, Bandung. We used NMDs and healthy patients to test the validity and reliability of the quantitative methodology. We added healthy patients as participants due to a minimum of NMDs participants were not being fulfilled. This study used purposive sampling, then included participants who understand *Bahasa Indonesia*, were aware and had a good ability to carry out the examination procedures instructed. The study participants' exclusion criteria were children with NMDs who have cognitive impairments and refuse to join this study.

### Statistical analysis

The data was collected from participants’ perceptions of the Indonesian version of the PedsQL™ 3.0 Neuromuscular Module statements. This study used the Rasch model with Winsteps software version 3.73 to measure the content validity and reliability. Rasch model can transform the categorical data from the PedsQL™ 3.0 Neuromuscular Module Indonesian Version (a 5-point Likert scale; 0 = never up to 4 = almost always) into interval data through logit units [11, 13, 14].

The reliability’s result contains information about the quality of the overall participant response pattern (Person Reliability), the quality of items used in the instrument (Item Reliability), and the interaction between the person and the items measured from the Cronbach Alpha value. In addition, construct validity, items’ validity, and rating scale’s validity of The PedsQL™ 3.0 Neuromuscular Module Indonesian Version were also measured using the Rasch model.

This study used healthy patients, so we performed a differential item functioning (DIF) analysis to confirm the suitability of the PedsQL™ 3.0 Neuromuscular Module Indonesian Version for NMDs patients (Additional file 1: Original and Indonesian version of the PedsQL™ 3.0 Neuromuscular Module).

### Time and place

We did the study from 1st January 2020 to 31st November 2020. Most of the data collection process was done online. Several participants were willing to be contacted face to face. We adjusted the meeting by their preferences and comfortability due to the coronavirus disease-2019 (COVID-19) pandemic.

### Ethics

This study's data collection process was carried out after obtaining a letter of ethical suitability number 223/UN6.KEP/EC/2020 from the Research Ethics Committee of the Faculty of Medicine, Universitas Padjadjaran, including applying the established COVID-19 Pandemic health protocols.

## Results

For the first phase of the cognitive debriefing, the instruments were distributed to 13 NMDs participants (including their parents), consisting of 6 people aged 2–4 years, 4 people aged 5–7 years, 3 people aged 8–12 years, and 1 person aged 13–18 years. The first phase of cognitive debriefing results showed that some questions were still challenging to understand by the participants, for example, ‘I get sores and/or rashes’, what does ‘feels stiff’ means, or what does ‘long times to bathe/shower’ means. Most of the participants did not understand those statements. The questions were difficult to understand or still need more reviews. Thus, we revised from the result of the first phase. Furthermore, the second phase of cognitive debriefing was required to be conducted.

The second phase of the cognitive debriefing involved 7 NMDs participants, consisting of one person aged 2–4 years, three people aged 5–7 years, three people aged 8–12 years, and one person aged 13–18. The results of the second phase of this cognitive debriefing showed that parents understood questions more quickly and were more certain (confident) in determining their choices. PedsQL™ 3.0 Neuromuscular Module in Indonesian version then composed as the final module (PedsQL™ 3.0 Neuromuscular Module Indonesian Version) to be used in the validity and reliability test.

We gave the PedsQL™ 3.0 Neuromuscular Module Indonesian Version to the participants for testing the validity and reliability. It consisted of 84 parents (all age groups; 46 NMDs patients and 38 healthy patients) and 71 children (ranging from 5 to 18 years old; 33 NMDs patients and 38 healthy patients).

The PedsQL™ Neuromuscular Module Indonesian Version's validity and reliability analysis were presented in Tables 1 and 2 using the Winsteps program. These tables can provide general information about the participants' answer patterns, the instruments' quality, and the participants’ relationship to the questionnaire's questions.

**Table 1:** Summary of Rasch measurement model on the PedsQL™ 3.0 Neuromuscular Module Indonesian Version parent proxy-report

Parameter (with quality criteria^a^)	The PedsQL™ 3.0 Neuromuscular Module Indonesian Version			
	Total scale	About My Child’s Neuromuscular Disease Subscale	Communication Subscale	About Our Family Resources Subscale
*Model fit: summary of item*				
Item mean in logits (criteria: 0.0 logits)	0.0, SD = 0.48	0.0, SD = 0.54	0.0, SD = 0.23	0.0, SD = 0.53
Item reliability (criteria: good 0.81–0.90; very good 0.91–0.94; excellent > 0.94)	0.93	0.94	0.15	0.90
Item separation (criteria: > 3)	3.61	3.99	0.42	3.07
Item strata separated = [(4 × separation index) + 1] / 3 (criteria: fair, 2–3; good, 3–4; very good, 4–5; excellent, > 5)	5.15 $\approx$ 5 levels	5.65 $\approx$ 5 levels	0.89 $\approx$ 1 level	4.43 $\approx$ 4 levels
Item misfit MNSQ range extremes (criteria: good 0.5–2.0; very good 0.7–1.4; excellent 0.77–1.3)	Infit: 0.68–1.41 Outfit: 0.51–1.81	Infit: 0.66–1.31 Outfit: 0.53–1.68	Infit: 0.66–1.49 Outfit: 0.63–1.35	Infit:0.72–1.45 Outfit: 0.72–1.35
Point measure correlation (criteria: 0.4–0.85)	0.36–0.76	0.36–0.81	0.89–0.93	0.75–0.85
*Model fit: summary of persons*				
Person mean in logits (criteria: 0.0 logits)	-1.13, SD = 1.31	-1.18, SD = 1.25	-1.63, SD = 2.42	-0.77, SD = 1.49
Person reliability (criteria: good 0.81–0.90; very good 0.91–0.94; excellent > 0.94)	0.84	0.80	0.63	0.77
Person separation (criteria: > 2)	2.32	1.99	1.31	1.84
Person strata separated = [(4 × separation index) + 1] / 3 (criteria: fair, 2–3; good, 3–4; very good, 4–5; excellent, > 5)	3.43 $\approx$ 4 levels	2.99 $\approx$ 3 levels	2.08 $\approx$ 2 levels	2.67 $\approx$ 3 levels
*Cronbach alpha* (criteria: fair 0.67–0.80; good 0.81–0.90; very good 0.91–0.94; excellent > 0.94)	0.95	0.93	0.91	0.87
*Dimensionality*				
Raw variance in data explained by measures (criteria > 40%)	51.9%	55.6%	55.6%	63.4%
Unexplained variance in 1st contrasts of PCA of residuals (criteria: fair, 10–15%; good, 5–10%; very good, 3–5%; excellent, < 3%)	8.4%	10.0%	29.6%	11.9%
*Rating scale analysis*				
Average observed (criteria: increases monotonically across rating scale)	Yes	NA	NA	NA
Outfit MNSQ (criteria: < 2 logits)	Yes	NA	NA	NA
Adjacent threshold distance (criteria: 1.4–5 logits)	(i) scale 0–1 was 0 to -0.09 = 0.09 logits; (ii) scale 1–2 was -0.09 to -0.79 = 0.70 logits; (iii) scale 2–3 was -0.79 to 0.71 = 1.50 logits; (iv) scale 3–4 was 0.71 to 0.17 = 0.54 logits	NA	NA	NA

SD: standard deviation; MNSQ: mean square; NA: not applicable

^a^Rating scale instrument quality criteria (Rasch Measurement Transactions 21, 1095, http://www.rasch.org/rmt/rmt211.pdf)

**Table 2:** Summary of Rasch measurement model on PedsQL™ 3.0 Neuromuscular Module Indonesian Version child self-report

Parameter (with quality criteria^a^)	The PedsQL™ 3.0 Neuromuscular Module Indonesian Version			
	Total scale	About My Child’s Neuromuscular Disease Subscale	Communication Subscale	About Our Family Resources Subscale
*Model fit: summary of item*				
Item mean in logits (criteria: 0.0 logits)	0.0, SD = 0.37	0.0, SD = 0.42	0.0, SD = 0.22	0.0, SD = 0.42
Item reliability (criteria: good 0.81–0.90; very good 0.91–0.94; excellent > 0.94)	0.81	0.90	0.00	0.77
Item separation (criteria: > 3)	2.06	3.02	0.00	1.81
Separate item strata = [(4 × separation index) + 1] / 3 (criteria: fair, 2–3; good, 3–4; very good, 4–5; excellent, > 5)	3.08 $\approx$ 3 levels	4.36 $\approx$ 4 levels	0.33 $\approx$ 0 level	2.75 $\approx$ 3 level
Item misfit MNSQ range extremes (criteria: good 0.5–2.0; very good 0.7–1.4; excellent 0.77–1.3)	Infit: 0.53–1.45 Outfit: 0.50–1.73	Infit: 0.74–1.34 Outfit: 0.61–1.58	Infit: 0.65–1.65 Outfit: 0.60–1.60	Infit: 0.81–1,29 Outfit: 0.82–1.13
Point measure correlation (criteria: 0.4–0.85)	0.31–0.73	0.37–0.75	0.82–0.90	0.71–0.81
*Model fit: summary of persons*				
Person mean in logits (criteria: 0.0 logits)	-1.12, SD = 0.92	-1.02, SD = 0.96	-2.34, SD = 1.83	-1.69, SD = 1.65
Person reliability (criteria: good 0.81–0.90; very good 0.91–0.94; excellent > 0.94)	0.81	0.74	0.36	0.64
Person separation (criteria: > 2)	2.08	1.68	0.74	1.34
Separate person strata = [(4 × separation index) + 1] / 3 (criteria: fair, 2–3; good, 3–4; very good, 4–5; excellent, > 5)	3.11 $\approx$ 3 levels	2.57 $\approx$ 3 levels	1.32 $\approx$ 1 level	2.12 $\approx$ 2 levels
*Cronbach alpha* (criteria: fair 0.67–0.80; good 0.81–0.90; very good 0.91–0.94; excellent > 0.94)	0.93	0.90	0.85	0.85
*Dimensionality*				
Raw variance in data explained by measures (criteria > 40%)	40.9%	45.0%	53.4%	57.5%
Unexplained variance in 1st contrasts of PCA of residuals (criteria: fair, 10–15%; good, 5–10%; very good, 3–5%; excellent, < 3%)	12.6%	13.7%	36.8%	17.3%
*Rating scale analysis*				
Average observed (criteria: increases monotonically across rating scale)	Yes	NA	NA	NA
Outfit MNSQ (criteria: < 2 logits)	Yes	NA	NA	NA
Adjacent threshold distance (criteria: 1.4–5 logits)	(i) scale 0–1 was 0 to 0.10 = 0.10 logits; (ii) scale 1–2 was 0.10 to -0.85 = 0.95 logits; (iii) scale 2–3 was -0.85 to 0.56 = 1.41 logits; (iv) scale 3–4 was 0.56 to 0.18 = 0.38 logits	NA	NA	NA

SD: standard deviation; MNSQ: mean square; NA: not applicable

^a^Rating scale instrument quality criteria (Rasch Measurement Transactions 21, 1095, http://www.rasch.org/rmt/rmt211.pdf)

Reliability shows how items and persons can be reproduced from existing data. Parent proxy-report and child self-report total scales of the PedsQL™ 3.0 Neuromuscular Module Indonesian Version show good to very good reliability. The parent proxy-report total scales of the PedsQL™ 3.0 Neuromuscular Module Indonesian Version shows the Cronbach’s alpha was 0.95 (subscales range 0.87–0.95), person reliability was 0.84 (subscales range 0.63–0.84), item reliability was 0.93 (subscales range 0.15–0.94), person separation was 2.32 (subscales range 1.31–2.32), item separation was 3.61 (subscales range 0.42–3.99), person strata separation was 4 levels (subscales range 2–4 levels), and item strata separation was 5 levels (subscales range 1–5 levels).

The child self-report total scales of the PedsQL™ 3.0 Neuromuscular Module Indonesian Version show the Cronbach's alpha was 0.93 (subscales range 0.85–0.93), person reliability was 0.81 (subscales range 0.36–0.81), item reliability was 0.81 (subscales range 0.00–0.90), person separation was 2.08 (subscales range 0.74–1.34), item separation was 2.06 (subscales range 0.00–3.02), person strata separation was 3 levels (subscales range 1–3 levels), and item strata separation was 3 levels (subscales range 0–4 levels).

Table 1 shows the construct validity of the PedsQL™ 3.0 Neuromuscular Module Indonesian Version parent proxy-report. It was substantiated by sizable explained variance by measures (51.9–63.4%) and unexplained variance in 1st contrasts (8.4–29.6%). As shown in Table 2, construct validity of the PedsQL™ 3.0 Neuromuscular Module Indonesian Version child self-report was substantiated by sizable explained variance by measures (40.9–57.5%) and unexplained variance in 1st contrasts (12.6–36.8%).

There were no misfit items in the parent proxy-report and child self-report identified by the Rasch models. Either parent proxy-report or child self-report for rating scale increases monotonically across rating scale. Still, the adjacent threshold distance (0.09–1.50 for parent and 0.10–1.41 for child) did not meet the ideal Rasch-Andrich threshold (1.4–5).

**Fig. 3:**
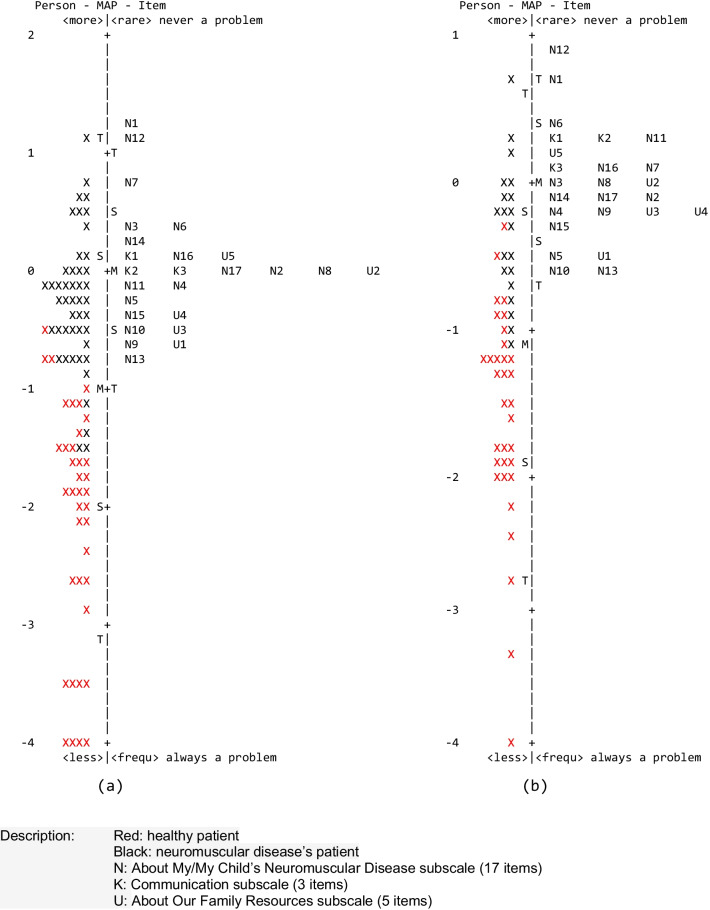
Person-Item Map (Wright Map) for **a** the PedsQL™ 3.0 Neuromuscular Module Indonesian Version parent proxy-report; **b** the PedsQL™ 3.0 Neuromuscular Module Indonesian Version child self-report. Description: Red: healthy patient, Black: neuromuscular disease’s patient, N: About My/My Child’s Neuromuscular Disease subscale (17 items), K: Communication subscale (3 items), U: About Our Family Resources subscale (5 items)

The person-item map for parent proxy-report (Fig. 3a) shows the items well-spread within two standard deviations (SD) of the item mean (0.0 logit). Although patients' perceptions varied, most were less likely to be bothered by neuromuscular disease problems (mean -1.13, SD = 1.31). The item that has never been a matter in the parent proxy-report was N1 (It is hard for my child to breathe). We also found that item N13 (It takes my child a long time to bathe or shower) was “almost always” becomes a matter.

Figure 3a shows the different responses between the parent of a healthy patient and an NMDs patient. Based on the questions asked in the instrument, almost all parents of healthy patients rated daily life problems rarely occur. Likewise, most parents of NMDs patients rated frequent daily life problems arise from the PedsQL™ Neuromuscular Module Indonesian Version.

In the child self-report of the PedsQL™ 3.0 Neuromuscular Module Indonesian Version, the person-item map (Fig. 3b) shows the items well-spread within two standard deviations (SD) of the item mean (0.0 logit). Although child perceptions varied, most were less likely to be bothered by neuromuscular disease problems (mean − 1.12, SD = 0.92). The item that has never been a matter in the child self-report was N12 (It is hard to swallow food). We also found that items N10 (It is hard to gain or lose when I want to) and N13 (It takes me a long time to bathe or shower) were “almost always” become a matter.

Figure 3b shows the different responses between healthy patients and NMDs patients. Based on the questions asked in the instrument, almost all healthy patients rated daily life problems rarely occur. Likewise, most NMDs patients rated frequent daily life problems from the PedsQL™ Neuromuscular Module Indonesian Version.

**Fig. 4:**
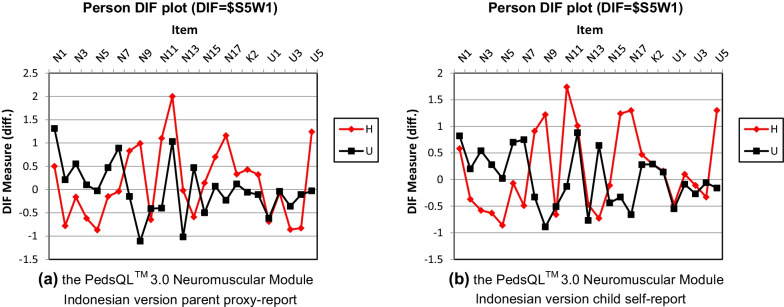
Differential Item Functioning (DIF) plot based on health status (H = healthy patients; U = unhealthy/neuromuscular disease’s patients; N = About My/My Child’s Neuromuscular Disease subscale (17 items); K = Communication subscale (3 items); U = About Our Family Resources subscale (5 items)). **a** The PedsQL™ 3.0 Neuromuscular Module Indonesian Version parent proxy-report. **b** The PedsQL™ 3.0 Neuromuscular Module Indonesian Version child self-report

There is a differential item functioning (DIF) if the difference of DIF measure between 2 groups is ‘greater than or equal 0.5’ or ‘less than or equal -0.5’. Figure 4a shows DIF between parents of NMDs patients and healthy patients. Almost all items are biased, except N10, K1, K2, K3, U1, and U2. As shown in Fig. 4b, there were also biased items between NMDs patients and healthy patients. Again, some items are biased, except N1, N10, N12, N13, N15, K1, K2, K3, U1, U2, U3, and U4.

## Discussion

This study shows that the PedsQL™ Neuromuscular Module Indonesian Version (total scale), either parent proxy-report (α = 0.95) and child self-report (α = 0.93), obtained a very good reliability value.The PedsQL™ Neuromuscular Module Indonesian Version can differentiate NMDs patients from healthy children. These findings are the same as the original version (English version), the Chinese translation, and the Thai translation's reliability estimates [8–10, 15].

### Validity and reliability of the total scale of the PedsQL™ Neuromuscular Module Indonesian Version

In parent proxy-report and child self-report, the total scale of the PedsQL™ Neuromuscular Module Indonesian Version, person reliability (0.84 and 0.81), item reliability (0.93 and 0.81), person separation (2.32 and 2.08), and item separation (3.61 and 2.06) show good to very good criteria using Rasch model analysis. Separation provides an overview of the distribution of items and persons involved in this instrument to reflect the number of items and persons’ strata [16].

Unidimensionality is an analysis of the Rasch model to evaluate whether the instrument developed can measure what should be measured (construct validity) [17]. According to Sumintono & Widhiarso, the minimum requirement for unidimensionality is 20%. It is even better if the value is more than 40%. The unexplained variance in 1st contrast should not exceed 15% (poor if > 15%) [17, 18]. Total scale of the PedsQL Neuromuscular Module Indonesian Version shows fair to good construct validity in parent proxy-report (raw variance 51.9%; unexplained variance in 1st contrasts 8.4%) and child self-report (raw variance 40.9%; unexplained variance in 1st contrasts 12.6%). It means this instrument (25 items) can measure what attributes should be measured, which is the quality of life for patients with NMDs in this case.

The item validity can be analyzed to see the items' suitability. The fit/misfit analysis of items used the outfit mean square (MNSQ), Z standard (ZSTD), and point (PT) measure correlation criteria. However, item elimination criteria are based on the analysis results that prove the items are inconsistent, namely two of the three criteria above which one of them is a negative PT Measure Correlation value [17]. Positive PT Measure Correlation indicates no item polarization or conflict between the item and the instrument being measured. The easiest thing is to evaluate the MNSQ Outfit value of each item. If the value is 1.5–2.0, then the item is not productive/less suitable for instrument measurement but does not decrease the instrument's validity. If the MNSQ Outfit value is more than 2.0, then it distorts/reduces the instrument's validity. If the MNSQ Outfit value is less than 0.5, it is less productive or suitable for instrument measurement, but it does not decrease its validity. There are various reasons for items to be a misfit. It could be because the number of participants is small, the variation of participants is not diverse (seen from the reliability index and separation), or the response pattern given is not consistent [16].

The validity of the items in the total scale parent proxy-report and child self-report of the PedsQL™ Neuromuscular Module Indonesian Version shows no misfit items. It means that items do not have a conflict with the attributes that should be measured, in this case, the quality of life of the patient with NMDs.

### Validity and reliability of the subscales of the PedsQL™ Neuromuscular Module Indonesian Version

Subscale 1 itself, which identifies NMDs condition, Cronbach’s alpha (0.93 and 0.90), person reliability (0.80 and 0.74), item reliability (0.94 and 0.90), person separation (1.99 and 1.68), and item separation (3.99 and 3.02), shows fair to good criteria in parent proxy-reports and child self-reports. In Thailand, subscale 1 also showed a good result (child and parent self-report α = 0.89) [10]. Meanwhile, child and parent self-report in China showed α = 0.87, which means subscale 1 was also good [9]. In the original English language in Spinal Muscular Atrophy (SMA) patients and Duchenne Muscular Dystrophy (DMD) patients, subscale 1 showed good reliability in child self-report and parent proxy-report [8, 15]. It shows that Subscale 1 can measure the evaluation results of therapy/interventions for pediatricians and/or physiatrists.

However, the quality criteria of item reliability (0.15 and 0.00), item separation (0.42 and 0.00), item strata separated (1 and 0), person reliability (0.63 and 0.36), person separation (1.31 and 0.74), and person strata separated (2 and 1) are poor in Subscale 2/Communication parent proxy-report and child self-report. In China, subscale 2 showed a good result (child α = 0.80, ICC = 0.49; parent α = 0.83, ICC = 0.76) [9].Meanwhile, in the Thailand study, subscale 2 was good (child α = 0.82, ICC = 0.35; parent α = 0.92, ICC = 0.63; parent–child agreement ICC = 0.24), except for the child proxy of test–retest reliability and parent–child agreement showed poor to a fair agreement [10]. The original English language in SMA patients (child α = 0.77, ICC = 0.63; parent α = 0.91, ICC = 0.82; parent–child agreement ICC = 0.41) showed that subscale 2 had a good result [8]. This result is probably affected by the characteristics of participants. We included all NMD patients in this study, same as in the Thailand study, while in China study was only for DMD patients.

In Subscale 3/About Our Family Resources, the quality criteria of person reliability (0.77 and 0.64), person separation (1.84 and 1.34), and person strata separated (3 and 2) are fair for parent proxy-report and poor for child self-report. Meanwhile, the quality criteria of item reliability (0.90 and 0.77), item separation (3.07 and 1.81), and item strata separated (4 and 3) are good for parent proxy-report and fair for child self-report. This finding is the same as the previous study in Thailand [10]. These fair and poor results for the child self-report may be caused by a lack of items in composing the subscale, not easy to understand, lack of information on items, or small sample size. Meanwhile, in China's study, child self-report (α = 0.73) and parent proxy-report (α = 0.85) showed good results [9]. The original English language in SMA patients showed that subscale 3 has a good result (child α = 0.82; parent α = 0.77) [8]. Our results are similar to the Thailand study that included all NMD patients.

The quality criteria of unexplained variance in 1st contrast of PCA of residual in subscale 2/Communication (29.6% in parent proxy-report and 36.8% in child self-report) and Subscale 3/ About Our Family Resources (17.3% in child self-proxy) show poor results. The items in Subscale 2 and Subscale 3 cannot measure the attribute that should be measured. These findings are the same as a result of a previous study, the Thailand language translation [10]. These results follow the item and person reliability in parent proxy-report and child self-report of Subscale 2 and Subscale 3 previously mentioned. Subscales that do not exceed the minimum criteria for reliability should only be considered for descriptive analysis.

### Item and person mapping

The person-item maps (also called Wright Maps) can determine the difficulty hierarchy of items that match the abilities or perceptions of participants in the PedsQL™ 3.0 Neuromuscular Module Indonesian Version. These maps allow the visual analysis of the relationship between persons and items' measures. Such as item redundancy (i.e., items at the same difficulty level), trait gaps (that may indicate the need for more items to fill the gaps), items order that match the prediction of the test author or users (i.e., construct validity), and targeting between the items and person (i.e., whether item difficulty range matches the sample person range). This person-item map does not affect the validity and reliability of the instrument [16–20]. The person-item maps show that the parent proxy-report and child self-report have different 'measuring abilities'. It is indicated by ‘the most never been a matter’ item between parent (N1) and child (N12) is different. This difference in perception between parents and children occurs not because of mistranslation but because these perceptions or opinions are relatively individual. From Fig. 3a, b, both the parents and healthy children mostly do not have the matter asked by the items from the PedsQL™ Neuromuscular Module Indonesian Version. Different responses from child self-reports and parent proxy-reports are not unique problems. It is consistently documented in many completed HRQoL instruments from children with and without chronic illness [21].

### Rating scales

The rating scale's validity parent proxy-report and child self-report of the PedsQL™ Neuromuscular Module Indonesian Version show increasing monotonically across rating scales. Still, the adjacent threshold distance did not meet the ideal Rasch-Andrich threshold. It means the potential of all these ratings from the Likert scale is not fully utilized by participants [16, 17]. It confirms that the participants cannot discriminate between the five rating units (0 = never a problem to 4 = almost always a problem). This result indicates that researchers (especially in Indonesia) cannot determine participants' quality of life whether or not they have a good quality of life based on the total score of this instrument because this rating scale is not perceived fairly by participants. Disordered Andrich thresholds do not interfere with the validity and reliability of the instrument, but they may impact the rating scale functions [22].

### Differential item functioning

Differential Item Functioning (DIF) indicates the items’ function, which acts differently for different sample group members. There are two groups of participants in this study, NMDs patients and healthy patients. DIF in the PedsQL™ Neuromuscular Module Indonesian Version indicates that these items function correctly because they can distinguish two groups of participants. However, if there is no DIF, the item does not function correctly because it cannot differentiate between them [17]. The items that do not function appropriately to distinguish between the two groups in the parent proxy-report of the PedsQL™ Neuromuscular Module Indonesian Version were N10 (It is hard for my child to gain or lose weight when he or she wants to) because parents' anxiety about their children's need to eat has similarities; K1 (It is hard for my child to tell the doctors and nurses how he or she feels), K2 (It is hard for my child to ask the doctors and nurses questions), K3 (It is hard for my child to explain his or her illness to other people), U1 (It is hard for our family to plan activities like vacations), and U2 (It is hard for our family to get enough rest).

Children with NMDs who participated in this study were selected who could follow instructions well and had no cognitive impairment. There was no difference in communication skills between the two groups. Communication skills in children develop according to their emotional development [23, 24].This study was conducted during the pandemic COVID-19, so U1 and U2 did not differ between the two groups. The two groups of participants cannot go on vacation due to the pandemic (social distancing) for the healthy group and physical condition for the NMDs group. This pandemic also has a psychological impact, such as depression, anxiety, and insomnia in all society levels [25–28]. Parents with healthy children may experience difficulty resting, possibly due to the effect of the pandemic [29–31]. Meanwhile, parents of children with NMDs find it difficult to rest because they care for their sick children.

The items that do not function appropriately to differentiate between the two groups in the child self-report of the PedsQL™ Neuromuscular Module Indonesian Version were N1 (It is hard to breath), N10 (It is hard to gain or lose weight when I want to), N12 (It is hard to swallow food), N13 (It takes me a long time to bathe or shower), N15 (I take a long time to eat), K1 (It is hard for me to tell the doctors and nurses how I feel), K2 (It is hard for me to ask the doctors and nurses questions), K3 (It is hard for me to explain my illness to other people), U1 (It is hard for my family to plan activities like vacations), U2 (It is hard for my family to get enough rest), U3 (I think money is a problem in our family), and U4 (I think my family has a lot of problems). It might be an indication that this module was not a self-rated instrument. Thus, it should be guided or accompanied by the interviewer for child self-report. A multicultural characteristic of Indonesian society, which differs from the original version, also might be another factor that influences participants while filling the instrument.

Thus, the PedsQL™ Neuromuscular Module Indonesian Version instrument is still relevant to the original PedsQL™ Neuromuscular Module instrument and is feasible to measure the quality of life of pediatric patients suffering from NMDs.

### Limitation of the study

We conducted this study during the COVID-19 pandemic, in which there was an activity restriction included doctor examinations for healthy and sick participants. Since the COVID-19 pandemic has emerged for six months, the study’s results might be affected because the instrument asked the participants to express their perceptions in the past month.

## Conclusions

We conclude that the PedsQL Neuromuscular Module Indonesian Version, both parent proxy-report and child self-report, are reliable and valid, so it is feasible to measure the quality of life of pediatric patients suffering from NMDs. This instrument could measure the success of programs/interventions for treating patients with NMDs if they were assisted when filling the instrument. We suggest further research involving more participants in a broader area. It would be better if it were done after the pandemic period has passed. We hope the total scale of the PedsQL Neuromuscular Module Indonesian Version can be used in a standardized manner for all regions of Indonesia.

## Supplementary Information

**Additional file 1:** Original and Indonesian version of the PedsQL™ 3.0 Neuromuscular Module.

## Data Availability

The data sets used and/or analyzed during the current study are available from the corresponding author on reasonable request.

## Abbreviations

CMT: Charcot Marie Tooth
COVID-19: Coronavirus disease 2019
CTT: Classical Test Theory
DIF: Differential Item Functioning
DMD: Duchenne Muscular Dystrophy
HRQoL: Health-related quality of life
ICC: Intraclass Correlation Coefficient
MNSQ: Mean square
NMDs: Neuromuscular diseases
PCA: Principal Component Analysis
PT: Point
SD: Standard deviations
SMA: Spinal Muscular Atrophy
ZSTD: Z standard
