# Supplementary material for: Translation and validation of Indonesian version of Pediatric Quality of Life Inventory™ (PedsQL™) Neuromuscular Module
Source: Health Qual Life Outcomes. 2022 Feb 24;20:33. doi: 10.1186/s12955-022-01933-x (PMC8867449; doi:10.1186/s12955-022-01933-x)
Supplement: Supplementary file 1 — Additional file 1: Original and Indonesian version of the PedsQL™ 3.0 Neuromuscular Module. [file 12955_2022_1933_MOESM1_ESM.pdf]

ID# ______________________

Date: ______________________

# PedsQL ™

## Neuromuscular Module

Version 3.0

**PARENT of TODDLER** (ages **2-4**)

**DIRECTIONS**

Children with neuromuscular disorders sometimes have special problems. Please tell us **how much of a problem** each one has been for your child during the **past ONE month** by circling:

**0** if it is **never** a problem
**1** if it is **almost never** a problem
**2** if it is **sometimes** a problem
**3** if it is **often** a problem
**4** if it is **almost always** a problem

There are no right or wrong answers.
If you do not understand a question, please ask for help.


Not to be reproduced without permission
Copyright © 1998 JW Varni, Ph.D.
All rights reserved

*In the past **ONE month**, how much of a **problem** has this been for your child …*

| **ABOUT MY CHILD'S NEUROMUSCULAR DISEASE** ***(problems with…)*** | **Never** | **Almost Never** | **Some-times** | **Often** | **Almost Always** |
|---|---|---|---|---|---|
| 1. It is hard for my child to breathe | 0 | 1 | 2 | 3 | 4 |
| 2. My child gets sick easily | 0 | 1 | 2 | 3 | 4 |
| 3. My child gets sores and/or rashes | 0 | 1 | 2 | 3 | 4 |
| 4. My child's legs hurt | 0 | 1 | 2 | 3 | 4 |
| 5. My child feels tired | 0 | 1 | 2 | 3 | 4 |
| 6. My child's back feels stiff | 0 | 1 | 2 | 3 | 4 |
| 7. My child wakes up tired | 0 | 1 | 2 | 3 | 4 |
| 8. My child's hands are weak | 0 | 1 | 2 | 3 | 4 |
| 9. It is hard for my child to use the bathroom | 0 | 1 | 2 | 3 | 4 |
| 10. It is hard for my child to gain or lose weight when he or she wants to | 0 | 1 | 2 | 3 | 4 |
| 11. It is hard for my child to use his or her hands | 0 | 1 | 2 | 3 | 4 |
| 12. It is hard for my child to swallow food | 0 | 1 | 2 | 3 | 4 |
| 13. It takes my child a long time to bathe or shower | 0 | 1 | 2 | 3 | 4 |
| 14. My child gets hurt accidentally | 0 | 1 | 2 | 3 | 4 |
| 15. My child takes a long time to eat | 0 | 1 | 2 | 3 | 4 |
| 16. It is hard for my child to turn him or herself during the night | 0 | 1 | 2 | 3 | 4 |
| 17. It is hard for my child to go places with his or her equipment | 0 | 1 | 2 | 3 | 4 |

| **COMMUNICATION** ***(problems with…)*** | **Never** | **Almost Never** | **Some-times** | **Often** | **Almost Always** |
|---|---|---|---|---|---|
| 1. It is hard for my child to tell the doctors and nurses how he or she feels | 0 | 1 | 2 | 3 | 4 |
| 2. It is hard for my child to ask the doctors and nurses questions | 0 | 1 | 2 | 3 | 4 |
| 3. It is hard for my child to explain his or her illness to other people | 0 | 1 | 2 | 3 | 4 |

| **ABOUT OUR FAMILY RESOURCES** ***(problems with…)*** | **Never** | **Almost Never** | **Some-times** | **Often** | **Almost Always** |
|---|---|---|---|---|---|
| 1. It is hard for our family to plan activities like vacations | 0 | 1 | 2 | 3 | 4 |
| 2. It is hard for our family to get enough rest | 0 | 1 | 2 | 3 | 4 |
| 3. I think money is a problem in our family | 0 | 1 | 2 | 3 | 4 |
| 4. I think our family has a lot of problems | 0 | 1 | 2 | 3 | 4 |
| 5. My child does not have the equipment he or she needs | 0 | 1 | 2 | 3 | 4 |


Not to be reproduced without permission
Copyright © 1998 JW Varni, Ph.D.
All rights reserved

 Copyright © 1998 JW Varni, Ph.D.

All rights reserved

ID# ____________________________

Date:__________________________

# PedsQL ™

## Neuromuscular Module

Version 3.0

**YOUNG CHILD REPORT** (ages **5-7**)

Instructions for interviewer:

***I am going to ask you some questions about things that might be a problem for some children. I want to know how much of a problem any of these things might be for you.***

Show the child the template and point to the responses as you read.

***If it is not at all a problem for you, point to the smiling face***

***If it is sometimes a problem for you, point to the middle face***

***If it is a problem for you a lot, point to the frowning face***

***I will read each question. Point to the pictures to show me how much of a problem it is for you. Let's try a practice one first.***

| | Not at all | Sometimes | A lot |
|---|---|---|---|
| **Is it hard for you to snap your fingers** | ☺ | 😐 | ☹ |

***Ask the child to demonstrate snapping his or her fingers to determine whether or not the question was answered correctly. Repeat the question if the child demonstrates a response that is different from his or her action.***


Not to be reproduced without permission

Copyright © 1998 JW Varni, Ph.D.
All rights reserved

***Think about how you have been doing for the last few weeks. Please listen carefully to each sentence and tell me how much of a problem this is for you.***

After reading the item, gesture to the template. If the child hesitates or does not seem to understand how to answer, read the response options while pointing at the faces.

| **ABOUT MY NEUROMUSCULAR DISEASE** *(problems with…)* | **Not at all** | **Some-times** | **Alot** |
|---|---|---|---|
| 1. It is hard to breathe | 0 | 2 | 4 |
| 2. I get sick easily | 0 | 2 | 4 |
| 3. I get sores and/or rashes | 0 | 2 | 4 |
| 4. My legs hurt | 0 | 2 | 4 |
| 5. I feel tired | 0 | 2 | 4 |
| 6. My back feels stiff | 0 | 2 | 4 |
| 7. I wake up tired | 0 | 2 | 4 |
| 8. My hands are weak | 0 | 2 | 4 |
| 9. It is hard to use the bathroom | 0 | 2 | 4 |
| 10. It is hard to gain or lose weight when I want to | 0 | 2 | 4 |
| 11. It is hard to use my hands | 0 | 2 | 4 |
| 12. It is hard to swallow food | 0 | 2 | 4 |
| 13. It takes me a long time to bathe or shower | 0 | 2 | 4 |
| 14. I get hurt accidentally | 0 | 2 | 4 |
| 15. I take a long time to eat | 0 | 2 | 4 |
| 16. It is hard to turn myself during the night | 0 | 2 | 4 |
| 17. It is hard for me to go places with my equipment | 0 | 2 | 4 |


Not to be reproduced without permission
Copyright © 1998 JW Varni, Ph.D.
All rights reserved

# How much of a problem is this for you?

| Not at all | Sometimes | A lot |
| --- | --- | --- |
| 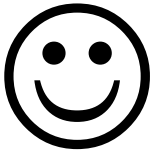 | 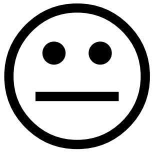 | 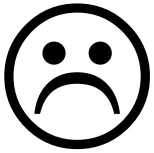 |



Not to be reproduced without permission

Copyright © 1998 JW Varni, Ph.D.
All rights reserved

ID# ____________________________

Date:___________________________

# PedsQL ™

## Neuromuscular Module

Version 3.0

**PARENT REPORT for YOUNG CHILD** (ages **5-7**)

**DIRECTIONS**

Children with neuromuscular disorders sometimes have special problems. Please tell us **how much of a problem** each one has been for your child during the **past ONE month** by circling:

**0** if it is **never** a problem
**1** if it is **almost never** a problem
**2** if it is **sometimes** a problem
**3** if it is **often** a problem
**4** if it is **almost always** a problem

There are no right or wrong answers.
If you do not understand a question, please ask for help.


Not to be reproduced without permission
Copyright © 1998 JW Varni, Ph.D.
All rights reserved

*In the past **ONE month**, how much of a **problem** has this been for your child …*

| **ABOUT MY CHILD'S NEUROMUSCULAR DISEASE** ***(problems with…)*** | **Never** | **Almost Never** | **Some-times** | **Often** | **Almost Always** |
|---|---|---|---|---|---|
| 1. It is hard for my child to breathe | 0 | 1 | 2 | 3 | 4 |
| 2. My child gets sick easily | 0 | 1 | 2 | 3 | 4 |
| 3. My child gets sores and/or rashes | 0 | 1 | 2 | 3 | 4 |
| 4. My child's legs hurt | 0 | 1 | 2 | 3 | 4 |
| 5. My child feels tired | 0 | 1 | 2 | 3 | 4 |
| 6. My child's back feels stiff | 0 | 1 | 2 | 3 | 4 |
| 7. My child wakes up tired | 0 | 1 | 2 | 3 | 4 |
| 8. My child's hands are weak | 0 | 1 | 2 | 3 | 4 |
| 9. It is hard for my child to use the bathroom | 0 | 1 | 2 | 3 | 4 |
| 10. It is hard for my child to gain or lose weight when he or she wants to | 0 | 1 | 2 | 3 | 4 |
| 11. It is hard for my child to use his or her hands | 0 | 1 | 2 | 3 | 4 |
| 12. It is hard for my child to swallow food | 0 | 1 | 2 | 3 | 4 |
| 13. It takes my child a long time to bathe or shower | 0 | 1 | 2 | 3 | 4 |
| 14. My child gets hurt accidentally | 0 | 1 | 2 | 3 | 4 |
| 15. My child takes a long time to eat | 0 | 1 | 2 | 3 | 4 |
| 16. It is hard for my child to turn him or herself during the night | 0 | 1 | 2 | 3 | 4 |
| 17. It is hard for my child to go places with his or her equipment | 0 | 1 | 2 | 3 | 4 |

| **COMMUNICATION** ***(problems with…)*** | **Never** | **Almost Never** | **Some-times** | **Often** | **Almost Always** |
|---|---|---|---|---|---|
| 1. It is hard for my child to tell the doctors and nurses how he or she feels | 0 | 1 | 2 | 3 | 4 |
| 2. It is hard for my child to ask the doctors and nurses questions | 0 | 1 | 2 | 3 | 4 |
| 3. It is hard for my child to explain his or her illness to other people | 0 | 1 | 2 | 3 | 4 |

| **ABOUT OUR FAMILY RESOURCES** ***(problems with…)*** | **Never** | **Almost Never** | **Some-times** | **Often** | **Almost Always** |
|---|---|---|---|---|---|
| 1. It is hard for our family to plan activities like vacations | 0 | 1 | 2 | 3 | 4 |
| 2. It is hard for our family to get enough rest | 0 | 1 | 2 | 3 | 4 |
| 3. I think money is a problem in our family | 0 | 1 | 2 | 3 | 4 |
| 4. I think our family has a lot of problems | 0 | 1 | 2 | 3 | 4 |
| 5. My child does not have the equipment he or she needs | 0 | 1 | 2 | 3 | 4 |


Not to be reproduced without permission
Copyright © 1998 JW Varni, Ph.D.
All rights reserved

ID# ____________________________

Date:___________________________

# PedsQL ™

## Neuromuscular Module

Version 3.0

**CHILD REPORT** (ages **8-12**)

**DIRECTIONS**

Children with neuromuscular disorders sometimes have special problems. Please tell us **how much of a problem** each one has been for you during the **past ONE month** by circling:

**0** if it is **never** a problem
**1** if it is **almost never** a problem
**2** if it is **sometimes** a problem
**3** if it is **often** a problem
**4** if it is **almost always** a problem

There are no right or wrong answers.
If you do not understand a question, please ask for help.


Not to be reproduced without permission

Copyright © 1998 JW Varni, Ph.D.
All rights reserved

*In the past **ONE month**, how much of a **problem** has this been for you …*

| **ABOUT MY NEUROMUSCULAR DISEASE** ***(problems with…)*** | **Never** | **Almost Never** | **Some-times** | **Often** | **Almost Always** |
|---|---|---|---|---|---|
| 1. It is hard to breathe | 0 | 1 | 2 | 3 | 4 |
| 2. I get sick easily | 0 | 1 | 2 | 3 | 4 |
| 3. I get sores and/or rashes | 0 | 1 | 2 | 3 | 4 |
| 4. My legs hurt | 0 | 1 | 2 | 3 | 4 |
| 5. I feel tired | 0 | 1 | 2 | 3 | 4 |
| 6. My back feels stiff | 0 | 1 | 2 | 3 | 4 |
| 7. I wake up tired | 0 | 1 | 2 | 3 | 4 |
| 8. My hands are weak | 0 | 1 | 2 | 3 | 4 |
| 9. It is hard to use the bathroom | 0 | 1 | 2 | 3 | 4 |
| 10. It is hard to gain or lose weight when I want to | 0 | 1 | 2 | 3 | 4 |
| 11. It is hard to use my hands | 0 | 1 | 2 | 3 | 4 |
| 12. It is hard to swallow food | 0 | 1 | 2 | 3 | 4 |
| 13. It takes me a long time to bathe or shower | 0 | 1 | 2 | 3 | 4 |
| 14. I get hurt accidentally | 0 | 1 | 2 | 3 | 4 |
| 15. I take a long time to eat | 0 | 1 | 2 | 3 | 4 |
| 16. It is hard to turn myself during the night | 0 | 1 | 2 | 3 | 4 |
| 17. It is hard for me to go places with my equipment | 0 | 1 | 2 | 3 | 4 |

| **COMMUNICATION** ***(problems with…)*** | **Never** | **Almost Never** | **Some-times** | **Often** | **Almost Always** |
|---|---|---|---|---|---|
| 1. It is hard for me to tell the doctors and nurses how I feel | 0 | 1 | 2 | 3 | 4 |
| 2. It is hard for me to ask the doctors and nurses questions | 0 | 1 | 2 | 3 | 4 |
| 3. It is hard for me to explain my illness to other people | 0 | 1 | 2 | 3 | 4 |

| **ABOUT OUR FAMILY RESOURCES** ***(problems with…)*** | **Never** | **Almost Never** | **Some-times** | **Often** | **Almost Always** |
|---|---|---|---|---|---|
| 1. It is hard for my family to plan activities like vacations | 0 | 1 | 2 | 3 | 4 |
| 2. It is hard for my family to get enough rest | 0 | 1 | 2 | 3 | 4 |
| 3. I think money is a problem in our family | 0 | 1 | 2 | 3 | 4 |
| 4. I think my family has a lot of problems | 0 | 1 | 2 | 3 | 4 |
| 5. I do not have the equipment I need | 0 | 1 | 2 | 3 | 4 |


Not to be reproduced without permission
Copyright © 1998 JW Varni, Ph.D.
All rights reserved

ID# ___________________________

Date: ___________________________

# PedsQL ™

## Neuromuscular Module

Version 3.0

**PARENT REPORT for CHILD** (ages **8-12**)

### DIRECTIONS

Children with neuromuscular disorders sometimes have special problems. Please tell us **how much of a problem** each one has been for your child during the **past ONE month** by circling:

**0** if it is **never** a problem
**1** if it is **almost never** a problem
**2** if it is **sometimes** a problem
**3** if it is **often** a problem
**4** if it is **almost always** a problem

There are no right or wrong answers.
If you do not understand a question, please ask for help.


Not to be reproduced without permission

Copyright © 1998 JW Varni, Ph.D.
All rights reserved

*In the past **ONE month**, how much of a **problem** has this been for your child …*

| **ABOUT MY CHILD'S NEUROMUSCULAR DISEASE** ***(problems with…)*** | **Never** | **Almost Never** | **Some-times** | **Often** | **Almost Always** |
|---|---|---|---|---|---|
| 1. It is hard for my child to breathe | 0 | 1 | 2 | 3 | 4 |
| 2. My child gets sick easily | 0 | 1 | 2 | 3 | 4 |
| 3. My child gets sores and/or rashes | 0 | 1 | 2 | 3 | 4 |
| 4. My child's legs hurt | 0 | 1 | 2 | 3 | 4 |
| 5. My child feels tired | 0 | 1 | 2 | 3 | 4 |
| 6. My child's back feels stiff | 0 | 1 | 2 | 3 | 4 |
| 7. My child wakes up tired | 0 | 1 | 2 | 3 | 4 |
| 8. My child's hands are weak | 0 | 1 | 2 | 3 | 4 |
| 9. It is hard for my child to use the bathroom | 0 | 1 | 2 | 3 | 4 |
| 10. It is hard for my child to gain or lose weight when he or she wants to | 0 | 1 | 2 | 3 | 4 |
| 11. It is hard for my child to use his or her hands | 0 | 1 | 2 | 3 | 4 |
| 12. It is hard for my child to swallow food | 0 | 1 | 2 | 3 | 4 |
| 13. It takes my child a long time to bathe or shower | 0 | 1 | 2 | 3 | 4 |
| 14. My child gets hurt accidentally | 0 | 1 | 2 | 3 | 4 |
| 15. My child takes a long time to eat | 0 | 1 | 2 | 3 | 4 |
| 16. It is hard for my child to turn him or herself during the night | 0 | 1 | 2 | 3 | 4 |
| 17. It is hard for my child to go places with his or her equipment | 0 | 1 | 2 | 3 | 4 |

| **COMMUNICATION** ***(problems with…)*** | **Never** | **Almost Never** | **Some-times** | **Often** | **Almost Always** |
|---|---|---|---|---|---|
| 1. It is hard for my child to tell the doctors and nurses how he or she feels | 0 | 1 | 2 | 3 | 4 |
| 2. It is hard for my child to ask the doctors and nurses questions | 0 | 1 | 2 | 3 | 4 |
| 3. It is hard for my child to explain his or her illness to other people | 0 | 1 | 2 | 3 | 4 |

| **ABOUT OUR FAMILY RESOURCES** ***(problems with…)*** | **Never** | **Almost Never** | **Some-times** | **Often** | **Almost Always** |
|---|---|---|---|---|---|
| 1. It is hard for our family to plan activities like vacations | 0 | 1 | 2 | 3 | 4 |
| 2. It is hard for our family to get enough rest | 0 | 1 | 2 | 3 | 4 |
| 3. I think money is a problem in our family | 0 | 1 | 2 | 3 | 4 |
| 4. I think our family has a lot of problems | 0 | 1 | 2 | 3 | 4 |
| 5. My child does not have the equipment he or she needs | 0 | 1 | 2 | 3 | 4 |


Not to be reproduced without permission
Copyright © 1998 JW Varni, Ph.D.
All rights reserved

ID# ____________________________

Date:___________________________

# PedsQL ™

## Neuromuscular Module

Version 3.0

**TEEN REPORT** (ages **13-18**)

**DIRECTIONS**

Teens with neuromuscular disorders sometimes have special problems. Please tell us **how much of a problem** each one has been for you during the **past ONE month** by circling:

**0** if it is **never** a problem
**1** if it is **almost never** a problem
**2** if it is **sometimes** a problem
**3** if it is **often** a problem
**4** if it is **almost always** a problem

There are no right or wrong answers.
If you do not understand a question, please ask for help.


Not to be reproduced without permission
Copyright © 1998 JW Varni, Ph.D.
All rights reserved

*In the past **ONE month**, how much of a **problem** has this been for you …*

| **ABOUT MY NEUROMUSCULAR DISEASE** ***(problems with…)*** | **Never** | **Almost Never** | **Some-times** | **Often** | **Almost Always** |
|---|---|---|---|---|---|
| 1. It is hard to breathe | 0 | 1 | 2 | 3 | 4 |
| 2. I get sick easily | 0 | 1 | 2 | 3 | 4 |
| 3. I get sores and/or rashes | 0 | 1 | 2 | 3 | 4 |
| 4. My legs hurt | 0 | 1 | 2 | 3 | 4 |
| 5. I feel tired | 0 | 1 | 2 | 3 | 4 |
| 6. My back feels stiff | 0 | 1 | 2 | 3 | 4 |
| 7. I wake up tired | 0 | 1 | 2 | 3 | 4 |
| 8. My hands are weak | 0 | 1 | 2 | 3 | 4 |
| 9. It is hard to use the bathroom | 0 | 1 | 2 | 3 | 4 |
| 10. It is hard to gain or lose weight when I want to | 0 | 1 | 2 | 3 | 4 |
| 11. It is hard to use my hands | 0 | 1 | 2 | 3 | 4 |
| 12. It is hard to swallow food | 0 | 1 | 2 | 3 | 4 |
| 13. It takes me a long time to bathe or shower | 0 | 1 | 2 | 3 | 4 |
| 14. I get hurt accidentally | 0 | 1 | 2 | 3 | 4 |
| 15. I take a long time to eat | 0 | 1 | 2 | 3 | 4 |
| 16. It is hard to turn myself during the night | 0 | 1 | 2 | 3 | 4 |
| 17. It is hard for me to go places with my equipment | 0 | 1 | 2 | 3 | 4 |

| **COMMUNICATION** ***(problems with…)*** | **Never** | **Almost Never** | **Some-times** | **Often** | **Almost Always** |
|---|---|---|---|---|---|
| 1. It is hard for me to tell the doctors and nurses how I feel | 0 | 1 | 2 | 3 | 4 |
| 2. It is hard for me to ask the doctors and nurses questions | 0 | 1 | 2 | 3 | 4 |
| 3. It is hard for me to explain my illness to other people | 0 | 1 | 2 | 3 | 4 |

| **ABOUT OUR FAMILY RESOURCES** ***(problems with…)*** | **Never** | **Almost Never** | **Some-times** | **Often** | **Almost Always** |
|---|---|---|---|---|---|
| 1. It is hard for my family to plan activities like vacations | 0 | 1 | 2 | 3 | 4 |
| 2. It is hard for my family to get enough rest | 0 | 1 | 2 | 3 | 4 |
| 3. I think money is a problem in our family | 0 | 1 | 2 | 3 | 4 |
| 4. I think my family has a lot of problems | 0 | 1 | 2 | 3 | 4 |
| 5. I do not have the equipment I need | 0 | 1 | 2 | 3 | 4 |


Not to be reproduced without permission
Copyright © 1998 JW Varni, Ph.D.
All rights reserved

ID# ___________________________

Date:__________________________

# PedsQL ™

## Neuromuscular Module

Version 3.0

**PARENT REPORT for TEEN** (ages **13-18**)

### DIRECTIONS

Teens with neuromuscular disorders sometimes have special problems. Please tell us **how much of a problem** each one has been for your teen during the **past ONE month** by circling:

**0** if it is **never** a problem
**1** if it is **almost never** a problem
**2** if it is **sometimes** a problem
**3** if it is **often** a problem
**4** if it is **almost always** a problem

There are no right or wrong answers.
If you do not understand a question, please ask for help.


Not to be reproduced without permission
Copyright © 1998 JW Varni, Ph.D.
All rights reserved

*In the past **ONE month**, how much of a **problem** has this been for your teen …*

| **ABOUT MY CHILD'S NEUROMUSCULAR DISEASE** ***(problems with…)*** | **Never** | **Almost Never** | **Some-times** | **Often** | **Almost Always** |
|---|---|---|---|---|---|
| 1. It is hard for my child to breathe | 0 | 1 | 2 | 3 | 4 |
| 2. My child gets sick easily | 0 | 1 | 2 | 3 | 4 |
| 3. My child gets sores and/or rashes | 0 | 1 | 2 | 3 | 4 |
| 4. My child's legs hurt | 0 | 1 | 2 | 3 | 4 |
| 5. My child feels tired | 0 | 1 | 2 | 3 | 4 |
| 6. My child's back feels stiff | 0 | 1 | 2 | 3 | 4 |
| 7. My child wakes up tired | 0 | 1 | 2 | 3 | 4 |
| 8. My child's hands are weak | 0 | 1 | 2 | 3 | 4 |
| 9. It is hard for my child to use the bathroom | 0 | 1 | 2 | 3 | 4 |
| 10. It is hard for my child to gain or lose weight when he or she wants to | 0 | 1 | 2 | 3 | 4 |
| 11. It is hard for my child to use his or her hands | 0 | 1 | 2 | 3 | 4 |
| 12. It is hard for my child to swallow food | 0 | 1 | 2 | 3 | 4 |
| 13. It takes my child a long time to bathe or shower | 0 | 1 | 2 | 3 | 4 |
| 14. My child gets hurt accidentally | 0 | 1 | 2 | 3 | 4 |
| 15. My child takes a long time to eat | 0 | 1 | 2 | 3 | 4 |
| 16. It is hard for my child to turn him or herself during the night | 0 | 1 | 2 | 3 | 4 |
| 17. It is hard for my child to go places with his or her equipment | 0 | 1 | 2 | 3 | 4 |

| **COMMUNICATION** ***(problems with…)*** | **Never** | **Almost Never** | **Some-times** | **Often** | **Almost Always** |
|---|---|---|---|---|---|
| 1. It is hard for my child to tell the doctors and nurses how he or she feels | 0 | 1 | 2 | 3 | 4 |
| 2. It is hard for my child to ask the doctors and nurses questions | 0 | 1 | 2 | 3 | 4 |
| 3. It is hard for my child to explain his or her illness to other people | 0 | 1 | 2 | 3 | 4 |

| **ABOUT OUR FAMILY RESOURCES** ***(problems with…)*** | **Never** | **Almost Never** | **Some-times** | **Often** | **Almost Always** |
|---|---|---|---|---|---|
| 1. It is hard for our family to plan activities like vacations | 0 | 1 | 2 | 3 | 4 |
| 2. It is hard for our family to get enough rest | 0 | 1 | 2 | 3 | 4 |
| 3. I think money is a problem in our family | 0 | 1 | 2 | 3 | 4 |
| 4. I think our family has a lot of problems | 0 | 1 | 2 | 3 | 4 |
| 5. My child does not have the equipment he or she needs | 0 | 1 | 2 | 3 | 4 |


Not to be reproduced without permission
Copyright © 1998 JW Varni, Ph.D.
All rights reserved

ID# ____________________________

Date:___________________________

# PedsQL ™

## Neuromuscular Module

Version 3.0

**YOUNG ADULT REPORT** (ages **18-25**)

**DIRECTIONS**

Young adults with neuromuscular disorders sometimes have special problems. Please tell us **how much of a problem** each one has been for you during the **past ONE month** by circling:

**0** if it is **never** a problem
**1** if it is **almost never** a problem
**2** if it is **sometimes** a problem
**3** if it is **often** a problem
**4** if it is **almost always** a problem

There are no right or wrong answers.
If you do not understand a question, please ask for help.


Not to be reproduced without permission

Copyright © 1998 JW Varni, Ph.D.
All rights reserved

*In the past **ONE month**, how much of a **problem** has this been for you …*

| **ABOUT MY NEUROMUSCULAR DISEASE** ***(problems with…)*** | **Never** | **Almost Never** | **Some-times** | **Often** | **Almost Always** |
|---|---|---|---|---|---|
| 1. It is hard to breathe | 0 | 1 | 2 | 3 | 4 |
| 2. I get sick easily | 0 | 1 | 2 | 3 | 4 |
| 3. I get sores and/or rashes | 0 | 1 | 2 | 3 | 4 |
| 4. My legs hurt | 0 | 1 | 2 | 3 | 4 |
| 5. I feel tired | 0 | 1 | 2 | 3 | 4 |
| 6. My back feels stiff | 0 | 1 | 2 | 3 | 4 |
| 7. I wake up tired | 0 | 1 | 2 | 3 | 4 |
| 8. My hands are weak | 0 | 1 | 2 | 3 | 4 |
| 9. It is hard to use the bathroom | 0 | 1 | 2 | 3 | 4 |
| 10. It is hard to gain or lose weight when I want to | 0 | 1 | 2 | 3 | 4 |
| 11. It is hard to use my hands | 0 | 1 | 2 | 3 | 4 |
| 12. It is hard to swallow food | 0 | 1 | 2 | 3 | 4 |
| 13. It takes me a long time to bathe or shower | 0 | 1 | 2 | 3 | 4 |
| 14. I get hurt accidentally | 0 | 1 | 2 | 3 | 4 |
| 15. I take a long time to eat | 0 | 1 | 2 | 3 | 4 |
| 16. It is hard to turn myself during the night | 0 | 1 | 2 | 3 | 4 |
| 17. It is hard for me to go places with my equipment | 0 | 1 | 2 | 3 | 4 |

| **COMMUNICATION** ***(problems with…)*** | **Never** | **Almost Never** | **Some-times** | **Often** | **Almost Always** |
|---|---|---|---|---|---|
| 1. It is hard for me to tell the doctors and nurses how I feel | 0 | 1 | 2 | 3 | 4 |
| 2. It is hard for me to ask the doctors and nurses questions | 0 | 1 | 2 | 3 | 4 |
| 3. It is hard for me to explain my illness to other people | 0 | 1 | 2 | 3 | 4 |

| **ABOUT OUR FAMILY RESOURCES** ***(problems with…)*** | **Never** | **Almost Never** | **Some-times** | **Often** | **Almost Always** |
|---|---|---|---|---|---|
| 1. It is hard for my family to plan activities like vacations | 0 | 1 | 2 | 3 | 4 |
| 2. It is hard for my family to get enough rest | 0 | 1 | 2 | 3 | 4 |
| 3. I think money is a problem in our family | 0 | 1 | 2 | 3 | 4 |
| 4. I think my family has a lot of problems | 0 | 1 | 2 | 3 | 4 |
| 5. I do not have the equipment I need | 0 | 1 | 2 | 3 | 4 |


Not to be reproduced without permission
Copyright © 1998 JW Varni, Ph.D.
All rights reserved

ID# ____________________________

Date:___________________________

# PedsQL ™

## Neuromuscular Module

Version 3.0

### PARENT REPORT for YOUNG ADULTS (ages 18-25)

**DIRECTIONS**

Young adults with neuromuscular disorders sometimes have special problems. Please tell us **how much of a problem** each one has been for your child during the **past ONE month** by circling:

**0** if it is **never** a problem
**1** if it is **almost never** a problem
**2** if it is **sometimes** a problem
**3** if it is **often** a problem
**4** if it is **almost always** a problem

There are no right or wrong answers.
If you do not understand a question, please ask for help.


Not to be reproduced without permission
Copyright © 1998 JW Varni, Ph.D.
All rights reserved

*In the past **ONE month**, how much of a **problem** has this been for your child…*

| **ABOUT MY CHILD'S NEUROMUSCULAR DISEASE** ***(problems with…)*** | **Never** | **Almost Never** | **Some-times** | **Often** | **Almost Always** |
|---|---|---|---|---|---|
| 1. It is hard for my child to breathe | 0 | 1 | 2 | 3 | 4 |
| 2. My child gets sick easily | 0 | 1 | 2 | 3 | 4 |
| 3. My child gets sores and/or rashes | 0 | 1 | 2 | 3 | 4 |
| 4. My child's legs hurt | 0 | 1 | 2 | 3 | 4 |
| 5. My child feels tired | 0 | 1 | 2 | 3 | 4 |
| 6. My child's back feels stiff | 0 | 1 | 2 | 3 | 4 |
| 7. My child wakes up tired | 0 | 1 | 2 | 3 | 4 |
| 8. My child's hands are weak | 0 | 1 | 2 | 3 | 4 |
| 9. It is hard for my child to use the bathroom | 0 | 1 | 2 | 3 | 4 |
| 10. It is hard for my child to gain or lose weight when he or she wants to | 0 | 1 | 2 | 3 | 4 |
| 11. It is hard for my child to use his or her hands | 0 | 1 | 2 | 3 | 4 |
| 12. It is hard for my child to swallow food | 0 | 1 | 2 | 3 | 4 |
| 13. It takes my child a long time to bathe or shower | 0 | 1 | 2 | 3 | 4 |
| 14. My child gets hurt accidentally | 0 | 1 | 2 | 3 | 4 |
| 15. My child takes a long time to eat | 0 | 1 | 2 | 3 | 4 |
| 16. It is hard for my child to turn him or herself during the night | 0 | 1 | 2 | 3 | 4 |
| 17. It is hard for my child to go places with his or her equipment | 0 | 1 | 2 | 3 | 4 |

| **COMMUNICATION** ***(problems with…)*** | **Never** | **Almost Never** | **Some-times** | **Often** | **Almost Always** |
|---|---|---|---|---|---|
| 1. It is hard for my child to tell the doctors and nurses how he or she feels | 0 | 1 | 2 | 3 | 4 |
| 2. It is hard for my child to ask the doctors and nurses questions | 0 | 1 | 2 | 3 | 4 |
| 3. It is hard for my child to explain his or her illness to other people | 0 | 1 | 2 | 3 | 4 |

| **ABOUT OUR FAMILY RESOURCES** ***(problems with…)*** | **Never** | **Almost Never** | **Some-times** | **Often** | **Almost Always** |
|---|---|---|---|---|---|
| 1. It is hard for our family to plan activities like vacations | 0 | 1 | 2 | 3 | 4 |
| 2. It is hard for our family to get enough rest | 0 | 1 | 2 | 3 | 4 |
| 3. I think money is a problem in our family | 0 | 1 | 2 | 3 | 4 |
| 4. I think our family has a lot of problems | 0 | 1 | 2 | 3 | 4 |
| 5. My child does not have the equipment he or she needs | 0 | 1 | 2 | 3 | 4 |


Not to be reproduced without permission
Copyright © 1998 JW Varni, Ph.D.
All rights reserved

Not to be reproduced without permission
Copyright © 1998 JW Varni, Ph.D.
All rights reserved

ID# ______________________

Date:______________________

# PedsQL ™

## Modul Neuromuskular

Versi 3.0

### LAPORAN ORANGTUA DENGAN BALITA (Usia 2-4)

**PETUNJUK**

Balita usia 2-4 tahun dengan gangguan neuromuskular terkadang mengalami masalah yang unik dalam kegiatan sehari-harinya. Tolong sampaikan pada kami **seberapa sering masalah/ kesulitan** tersebut dialami anak Anda selama **SATU bulan terakhir**, dengan melingkari angka berikut ini untuk setiap masalah/ kesulitan yang dialami:

**0** jika **tidak pernah** dialami
**1** jika **hampir tidak pernah** dialami
**2** jika **kadang-kadang** dialami
**3** jika **sering** dialami
**4** jika **hampir selalu** dialami

Tidak ada jawaban yang benar atau salah.
Jika Anda tidak mengerti suatu pertanyaan, silahkan minta bantuan.


Tidak untuk diperbanyak tanpa ijin
Copyright © 1998 JW Varni, Ph.D.
All rights reserved

*Dalam **SATU bulan** terakhir, seberapa sering **Masalah/Kesulitan** berikut ini dialami oleh anak Anda …*

| | **Masalah/ Kesulitan yang berhubungan dengan : Penyakit Neuromuskuler Anak Saya** | **Tidak pernah** | **Hampir tidak pernah** | **Kadang-kadang** | **Sering** | **Hampir Selalu** |
|---|---|---|---|---|---|---|
| 1 | Kesulitan bernapas pada anak saya | 0 | 1 | 2 | 3 | 4 |
| 2 | Anak saya mudah sakit | 0 | 1 | 2 | 3 | 4 |
| 3 | Munculnya luka dan/ atau kemerahan pada anak saya | 0 | 1 | 2 | 3 | 4 |
| 4 | Nyeri pada kaki anak saya | 0 | 1 | 2 | 3 | 4 |
| 5 | Kecapean (kelelahan fisik) yang dirasakan anak saya | 0 | 1 | 2 | 3 | 4 |
| 6 | Rasa kaku pada punggung anak saya | 0 | 1 | 2 | 3 | 4 |
| 7 | Anak saya bangun tidur dalam keadaan letih | 0 | 1 | 2 | 3 | 4 |
| 8 | Kelemahan pada kedua tangan anak saya | 0 | 1 | 2 | 3 | 4 |
| 9 | Sulit untuk anak saya menggunakan kamar mandi | 0 | 1 | 2 | 3 | 4 |
| 10 | Sulit untuk anak saya menambah atau menurunkan berat badan saat dia menginginkannya | 0 | 1 | 2 | 3 | 4 |
| 11 | Sulit untuk anak saya menggunakan kedua tangannya | 0 | 1 | 2 | 3 | 4 |
| 12 | Sulit untuk anak saya menelan makanan | 0 | 1 | 2 | 3 | 4 |
| 13 | Anak saya membutuhkan waktu yang lebih lama untuk mandi atau menggunakan pancuran (*shower*) | 0 | 1 | 2 | 3 | 4 |
| 14 | Anak saya terluka secara tidak sengaja | 0 | 1 | 2 | 3 | 4 |
| 15 | Anak saya perlu waktu yang lebih lama untuk makan | 0 | 1 | 2 | 3 | 4 |
| 16 | Sulit untuk anak saya membalikkan badannya saat tidur di malam hari | 0 | 1 | 2 | 3 | 4 |
| 17 | Sulit untuk anak saya bepergian ke berbagai tempat dengan alat bantunya | 0 | 1 | 2 | 3 | 4 |

| | **Masalah/ Kesulitan yang berhubungan dengan : Komunikasi** | **Tidak pernah** | **Hampir tidak pernah** | **Kadang-kadang** | **Sering** | **Hampir Selalu** |
|---|---|---|---|---|---|---|
| 1 | Sulit untuk anak saya menyampaikan apa yang ia rasakan kepada dokter dan perawat. | 0 | 1 | 2 | 3 | 4 |
| 2 | Sulit untuk anak saya bertanya kepada dokter dan perawat. | 0 | 1 | 2 | 3 | 4 |
| 3 | Sulit untuk anak saya menjelaskan penyakitnya kepada orang lain. | 0 | 1 | 2 | 3 | 4 |

| | **Masalah/ Kesulitan yang berhubungan dengan : Keuangan Keluarga** | **Tidak pernah** | **Hampir tidak pernah** | **Kadang-kadang** | **Sering** | **Hampir Selalu** |
|---|---|---|---|---|---|---|
| 1 | Sulit untuk keluarga kami merencanakan kegiatan-kegiatan seperti liburan | 0 | 1 | 2 | 3 | 4 |
| 2 | Sulit untuk keluarga kami mendapatkan istirahat yang cukup | 0 | 1 | 2 | 3 | 4 |
| 3 | Menurut saya, keluarga kami mempunyai masalah dengan keuangan | 0 | 1 | 2 | 3 | 4 |
| 4 | Menurut saya, keluarga kami memiliki banyak masalah | 0 | 1 | 2 | 3 | 4 |
| 5 | Anak saya tidak memiliki peralatan/ alat bantu yang ia butuhkan | 0 | 1 | 2 | 3 | 4 |


Tidak untuk diperbanyak tanpa ijin
Copyright © 1998 JW Varni, Ph.D.
All rights reserved

ID# ____________________________

Date:___________________________

# PedsQL ™

## Modul Neuromuskular

Versi 3.0

### LAPORAN ANAK KECIL (Usia 5-7)

Petunjuk untuk pewawancara:

***Saya akan mengajukan beberapa pertanyaan tentang beberapa hal yang mungkin menjadi masalah bagi anak seumur kamu. Saya ingin tahu seberapa sering masalah tersebut kamu alami.***

Perlihatkan kepada anak pilihan gambar wajah dan tunjuk gambar wajah yang sesuai untuk setiap pertanyaan yang Saya bacakan.

***Jika yang dibacakan <u>sama sekali tidak</u> menjadi masalah untukmu, tunjuklah wajah yang tersenyum.***

***Jika yang dibacakan <u>kadang-kadang</u> menjadi masalah untukmu, tunjuklah wajah yang berada di tengah.***

***Jika yang dibacakan <u>sering</u> menjadi masalah untukmu, tunjuklah wajah yang cemberut.***

***Saya akan membacakan setiap pertanyaan. Tunjuklah gambar wajahnya untuk menunjukkan kepada saya seberapa sering masalah ini kamu alami. Mari kita coba satu dulu***

| | Tidak Sama sekali | Kadang-kadang | Sering |
|---|---|---|---|
| **Apakah sulit untuk kamu menjentikkan jari jemari** | ☺ | 😐 | ☹ |

***Mintalah anak tersebut untuk menjentikkan jari jemarinya untuk menentukan apakah pertanyaan diatas dijawab dengan benar atau tidak. Ulangi pertanyaannya jika anak tersebut tidak memberikan respon sesuai tindakan yang ditunjukkannya.***


Tidak untuk diperbanyak tanpa ijin

Copyright © 1998 JW Varni, Ph.D.
All rights reserved

***Pikirkan tentang apa yang Kamu alami selama beberapa minggu terakhir. Tolong dengarkanlah baik-baik setiap kalimat yang saya bacakan dan beri tahu saya seberapa sering hal tersebut menjadi masalah untukmu.***

Setelah membacakan kalimat tersebut, alihkan anak ke gambar wajah. Jika anak tampak ragu-ragu atau tidak memahami cara menjawabnya, bacakan pilihan jawabannya sambil menunjukkan gambar-gambar wajahnya.

| **Masalah yang berhubungan dengan :**<br>**Penyakit Neuromuskuler Saya** | **Tidak sama sekali** | **Kadang-kadang** | **Sering** |
|---|---|---|---|
| 1. Sulit bernafas | 0 | 2 | 4 |
| 2. Saya mudah sakit | 0 | 2 | 4 |
| 3. Saya mendapat luka dan / atau kemerahan | 0 | 2 | 4 |
| 4. Saya merasakan nyeri pada kaki saya | 0 | 2 | 4 |
| 5. Saya merasakan kecapean (kelelahan fisik) | 0 | 2 | 4 |
| 6. Saya merasakan kaku pada punggung saya | 0 | 2 | 4 |
| 7. Saya merasa letih ketika bangun tidur | 0 | 2 | 4 |
| 8. Kedua tangan saya lemah | 0 | 2 | 4 |
| 9. Saya sulit untuk menggunakan kamar mandi | 0 | 2 | 4 |
| 10. Saya sulit untuk menambah atau menurunkan berat badan saat saya menginginkannya | 0 | 2 | 4 |
| 11. Saya sulit menggunakan kedua tangan saya | 0 | 2 | 4 |
| 12. Saya sulit menelan makanan | 0 | 2 | 4 |
| 13. Saya perlu waktu yang lebih lama untuk mandi atau menggunakan pancuran (*shower*) | 0 | 2 | 4 |
| 14. Saya terluka secara tidak sengaja | 0 | 2 | 4 |
| 15. Saya perlu waktu yang lebih lama untuk makan | 0 | 2 | 4 |
| 16. Saya sulit membalikkan badan saat tidur di malam hari | 0 | 2 | 4 |
| 17. Saya sulit bepergian ke berbagai tempat dengan alat bantu saya | 0 | 2 | 4 |


Tidak untuk diperbanyak tanpa ijin
Copyright © 1998 JW Varni, Ph.D.
All rights reserved

# Seberapa sering ini menjadi masalah untukmu?

| Tidak sama sekali | Kadang-kadang | Sering |
|---|---|---|
| ☺ | 😐 | ☹ |



Tidak untuk diperbanyak tanpa ijin

Copyright © 1998 JW Varni, Ph.D.
All rights reserved

ID# ___________________________

Date:___________________________

# PedsQL ™

## Modul Neuromuskular

Versi 3.0

**LAPORAN ORANGTUA DENGAN ANAK KECIL (Usia 5-7)**

**PETUNJUK**

Anak kecil usia 5-7 tahun dengan gangguan neuromuskular terkadang mengalami masalah yang unik dalam kegiatan sehari-harinya. Tolong sampaikan pada kami **seberapa sering masalah/ kesulitan** tersebut dialami anak Anda selama **SATU bulan terakhir**, dengan melingkari angka berikut ini untuk setiap masalah/ kesulitan yang dialami:

**0** jika **tidak pernah** dialami
**1** jika **hampir tidak pernah** dialami
**2** jika **kadang-kadang** dialami
**3** jika **sering** dialami
**4** jika **hampir selalu** dialami

Tidak ada jawaban benar atau salah.
Jika Anda tidak mengerti suatu pertanyaan, silahkan minta bantuan.



Tidak untuk diperbanyak tanpa ijin

Copyright © 1998 JW Varni, Ph.D.
All rights reserved

*Dalam **SATU bulan** terakhir, seberapa sering **Masalah/Kesulitan** berikut ini dialami oleh anak Anda …*

| | **Masalah/ Kesulitan yang berhubungan dengan: Penyakit Neuromuskuler Anak Saya** | **Tidak pernah** | **Hampir tidak pernah** | **Kadang-kadang** | **Sering** | **Hampir Selalu** |
|---|---|---|---|---|---|---|
| 1 | Kesulitan bernapas pada anak saya | 0 | 1 | 2 | 3 | 4 |
| 2 | Anak saya mudah sakit | 0 | 1 | 2 | 3 | 4 |
| 3 | Munculnya luka dan/ atau kemerahan pada anak saya | 0 | 1 | 2 | 3 | 4 |
| 4 | Nyeri pada kaki anak saya | 0 | 1 | 2 | 3 | 4 |
| 5 | Kecapean (kelelahan fisik) yang dirasakan anak saya | 0 | 1 | 2 | 3 | 4 |
| 6 | Rasa kaku pada punggung anak saya | 0 | 1 | 2 | 3 | 4 |
| 7 | Anak saya bangun tidur dalam keadaan letih | 0 | 1 | 2 | 3 | 4 |
| 8 | Kelemahan pada kedua tangan anak saya | 0 | 1 | 2 | 3 | 4 |
| 9 | Sulit untuk anak saya menggunakan kamar mandi | 0 | 1 | 2 | 3 | 4 |
| 10 | Sulit untuk anak saya menambah atau menurunkan berat badan saat dia menginginkannya | 0 | 1 | 2 | 3 | 4 |
| 11 | Sulit untuk anak saya menggunakan kedua tangannya | 0 | 1 | 2 | 3 | 4 |
| 12 | Sulit untuk anak saya menelan makanan | 0 | 1 | 2 | 3 | 4 |
| 13 | Anak saya membutuhkan waktu yang lebih lama untuk mandi atau menggunakan pancuran (*shower*) | 0 | 1 | 2 | 3 | 4 |
| 14 | Anak saya terluka secara tidak sengaja | 0 | 1 | 2 | 3 | 4 |
| 15 | Anak saya perlu waktu yang lebih lama untuk makan | 0 | 1 | 2 | 3 | 4 |
| 16 | Sulit untuk anak saya membalikkan badannya saat tidur di malam hari | 0 | 1 | 2 | 3 | 4 |
| 17 | Sulit untuk anak saya bepergian ke berbagai tempat dengan alat bantunya | 0 | 1 | 2 | 3 | 4 |

| | **Masalah/ Kesulitan yang berhubungan dengan : Komunikasi** | **Tidak pernah** | **Hampir tidak pernah** | **Kadang-kadang** | **Sering** | **Hampir Selalu** |
|---|---|---|---|---|---|---|
| 1 | Sulit untuk anak saya menyampaikan apa yang ia rasakan kepada dokter dan perawat. | 0 | 1 | 2 | 3 | 4 |
| 2 | Sulit untuk anak saya bertanya kepada dokter dan perawat. | 0 | 1 | 2 | 3 | 4 |
| 3 | Sulit untuk anak saya menjelaskan penyakitnya kepada orang lain. | 0 | 1 | 2 | 3 | 4 |

| | **Masalah/ Kesulitan yang berhubungan dengan : Keuangan Keluarga** | **Tidak pernah** | **Hampir tidak pernah** | **Kadang-kadang** | **Sering** | **Hampir Selalu** |
|---|---|---|---|---|---|---|
| 1 | Sulit untuk keluarga kami merencanakan kegiatan-kegiatan seperti liburan | 0 | 1 | 2 | 3 | 4 |
| 2 | Sulit untuk keluarga kami mendapatkan istirahat yang cukup | 0 | 1 | 2 | 3 | 4 |
| 3 | Menurut saya, keluarga kami mempunyai masalah dengan keuangan | 0 | 1 | 2 | 3 | 4 |
| 4 | Menurut saya, keluarga kami memiliki banyak masalah | 0 | 1 | 2 | 3 | 4 |
| 5 | Anak saya tidak memiliki peralatan/ alat bantu yang ia butuhkan | 0 | 1 | 2 | 3 | 4 |


Tidak untuk diperbanyak tanpa ijin
Copyright © 1998 JW Varni, Ph.D.
All rights reserved

Copyright © 1998 JW Varni, Ph.D.
All rights reserved

ID# ___________________________

Date:___________________________

# PedsQL ™

## Modul Neuromuskular

Versi 3.0

**LAPORAN ANAK (Usia 8-12)**

**PETUNJUK**

Anak-anak dengan gangguan neuromuskuler seusia kamu terkadang mengalami masalah yang unik dalam kegiatan sehari-hari. Tolong ceritakan kepada kami **seberapa sering** kamu mengalami **masalah/kesulitan** tersebut dalam **SATU bulan terakhir** ini dengan melingkari salah satu angka berikut untuk setiap masalah/kesulitan yang disampaikan:

**0** Jika **tidak pernah** kamu alami
**1** Jika **hampir tidak pernah** kamu alami
**2** Jika **kadang-kadang** kamu alami
**3** Jika **sering** kamu alami
**4** Jika **hampir selalu** kamu alami

Tidak ada pilihan jawaban yang benar atau salah..
Jika ada pertanyaan yang kamu tidak mengerti, silakan minta bantuan


Tidak untuk diperbanyak tanpa ijin
Copyright © 1998 JW Varni, Ph.D.
All rights reserved

*Dalam **SATU bulan** terakhir, seberapa sering **Masalah/Kesulitan** di bawah ini kamu alami…*

| **Masalah/ Kesulitan yang berhubungan dengan: Penyakit Neuromuskuler Saya** | **Tidak pernah** | **Hampir tidak pernah** | **Kadang-kadang** | **Sering** | **Hampir Selalu** |
|---|---|---|---|---|---|
| 1 Sulit bernapas | 0 | 1 | 2 | 3 | 4 |
| 2 Saya mudah sakit | 0 | 1 | 2 | 3 | 4 |
| 3 Saya mendapat luka dan / atau kemerahan | 0 | 1 | 2 | 3 | 4 |
| 4 Saya merasakan nyeri pada kaki saya | 0 | 1 | 2 | 3 | 4 |
| 5 Saya merasakan kecapean (kelelahan fisik) | 0 | 1 | 2 | 3 | 4 |
| 6 Saya merasakan kaku pada punggung | 0 | 1 | 2 | 3 | 4 |
| 7 Saya merasa letih ketika bangun tidur | 0 | 1 | 2 | 3 | 4 |
| 8 Kedua tangan saya lemah | 0 | 1 | 2 | 3 | 4 |
| 9 Saya sulit untuk menggunakan kamar mandi | 0 | 1 | 2 | 3 | 4 |
| 10 Saya sulit untuk menambah atau menurunkan berat badan saat saya menginginkannya | 0 | 1 | 2 | 3 | 4 |
| 11 Saya sulit menggunakan kedua tangan saya | 0 | 1 | 2 | 3 | 4 |
| 12 Saya sulit untuk menelan makanan | 0 | 1 | 2 | 3 | 4 |
| 13 Saya perlu waktu yang lebih lama untuk mandi atau menggunakan pancuran (*shower*) | 0 | 1 | 2 | 3 | 4 |
| 14 Saya terluka secara tidak sengaja | 0 | 1 | 2 | 3 | 4 |
| 15 Saya perlu waktu yang lebih lama untuk makan | 0 | 1 | 2 | 3 | 4 |
| 16 Saya sulit membalikkan badan saat tidur di malam hari | 0 | 1 | 2 | 3 | 4 |
| 17 Saya sulit bepergian ke berbagai tempat dengan alat bantu saya | 0 | 1 | 2 | 3 | 4 |

| **Masalah/ Kesulitan yang berhubungan dengan : Komunikasi** | **Tidak pernah** | **Hampir tidak pernah** | **Kadang-kadang** | **Sering** | **Hampir Selalu** |
|---|---|---|---|---|---|
| 1 Sulit untuk saya menyampaikan apa yang saya rasakan kepada dokter dan perawat. | 0 | 1 | 2 | 3 | 4 |
| 2 Sulit untuk saya bertanya kepada dokter dan perawat. | 0 | 1 | 2 | 3 | 4 |
| 3 Sulit untuk saya menjelaskan penyakit saya kepada orang lain. | 0 | 1 | 2 | 3 | 4 |

| **Masalah/ Kesulitan yang berhubungan dengan : Keuangan Keluarga Saya** | **Tidak pernah** | **Hampir tidak pernah** | **Kadang-kadang** | **Sering** | **Hampir Selalu** |
|---|---|---|---|---|---|
| 1 Sulit untuk keluarga saya merencanakan kegiatan-kegiatan seperti liburan | 0 | 1 | 2 | 3 | 4 |
| 2 Sulit untuk keluarga saya mendapatkan istirahat yang cukup | 0 | 1 | 2 | 3 | 4 |
| 3 Menurut saya, keluarga saya mempunyai masalah dengan keuangan | 0 | 1 | 2 | 3 | 4 |
| 4 Menurut saya, keluarga saya memiliki banyak masalah | 0 | 1 | 2 | 3 | 4 |
| 5 Saya tidak memiliki peralatan/ alat bantu yang saya butuhkan | 0 | 1 | 2 | 3 | 4 |


Tidak untuk diperbanyak tanpa ijin
Copyright © 1998 JW Varni, Ph.D.
All rights reserved

ID# ____________________________

Date:___________________________

# PedsQL ™

## Modul Neuromuskular

Versi 3.0

**LAPORAN ORANGTUA DENGAN ANAK (Usia 8-12)**

**PETUNJUK**

Anak usia 8-12 tahun dengan gangguan neuromuskular terkadang mengalami masalah yang unik dalam kegiatan sehari-harinya. Tolong sampaikan pada kami **seberapa sering masalah/ kesulitan** tersebut dialami anak Anda selama **SATU bulan terakhir**, dengan melingkari angka berikut ini untuk setiap masalah/ kesulitan yang dialami:

**0** jika **tidak pernah** dialami
**1** jika **hampir tidak pernah** dialami
**2** jika **kadang-kadang** dialami
**3** jika **sering** dialami
**4** jika **hampir selalu** dialami

Tidak ada jawaban benar atau salah.
Jika Anda tidak mengerti suatu pertanyaan, silahkan minta bantuan.


Tidak untuk diperbanyak tanpa ijin

Copyright © 1998 JW Varni, Ph.D.
All rights reserved

*Dalam **SATU bulan** terakhir, seberapa sering **Masalah/Kesulitan** berikut ini dialami oleh anak Anda …*

| | **Masalah/ Kesulitan yang berhubungan dengan: Penyakit Neuromuskuler Anak Saya** | **Tidak pernah** | **Hampir tidak pernah** | **Kadang-kadang** | **Sering** | **Hampir Selalu** |
|---|---|---|---|---|---|---|
| 1 | Kesulitan bernapas pada anak saya | 0 | 1 | 2 | 3 | 4 |
| 2 | Anak saya mudah sakit | 0 | 1 | 2 | 3 | 4 |
| 3 | Munculnya luka dan/ atau kemerahan pada anak saya | 0 | 1 | 2 | 3 | 4 |
| 4 | Nyeri pada kaki anak saya | 0 | 1 | 2 | 3 | 4 |
| 5 | Kecapean (kelelahan fisik) yang dirasakan anak saya | 0 | 1 | 2 | 3 | 4 |
| 6 | Rasa kaku pada punggung anak saya | 0 | 1 | 2 | 3 | 4 |
| 7 | Anak saya bangun tidur dalam keadaan letih | 0 | 1 | 2 | 3 | 4 |
| 8 | Kelemahan pada kedua tangan anak saya | 0 | 1 | 2 | 3 | 4 |
| 9 | Sulit untuk anak saya menggunakan kamar mandi | 0 | 1 | 2 | 3 | 4 |
| 10 | Sulit untuk anak saya menambah atau menurunkan berat badan saat dia menginginkannya | 0 | 1 | 2 | 3 | 4 |
| 11 | Sulit untuk anak saya untuk menggunakan kedua tangannya | 0 | 1 | 2 | 3 | 4 |
| 12 | Sulit untuk anak saya menelan makanan | 0 | 1 | 2 | 3 | 4 |
| 13 | Anak saya membutuhkan waktu yang lebih lama untuk mandi atau menggunakan pancuran (*shower*) | 0 | 1 | 2 | 3 | 4 |
| 14 | Anak saya terluka secara tidak sengaja | 0 | 1 | 2 | 3 | 4 |
| 15 | Anak saya perlu waktu yang lebih lama untuk makan | 0 | 1 | 2 | 3 | 4 |
| 16 | Sulit untuk anak saya membalikkan badannya saat tidur di malam hari | 0 | 1 | 2 | 3 | 4 |
| 17 | Sulit untuk anak saya bepergian ke berbagai tempat dengan alat bantunya | 0 | 1 | 2 | 3 | 4 |

| | **Masalah/ Kesulitan yang berhubungan dengan : Komunikasi** | **Tidak pernah** | **Hampir tidak pernah** | **Kadang-kadang** | **Sering** | **Hampir Selalu** |
|---|---|---|---|---|---|---|
| 1 | Sulit untuk anak saya menyampaikan apa yang ia rasakan kepada dokter dan perawat. | 0 | 1 | 2 | 3 | 4 |
| 2 | Sulit untuk anak saya bertanya kepada dokter dan perawat. | 0 | 1 | 2 | 3 | 4 |
| 3 | Sulit untuk anak saya menjelaskan penyakitnya kepada orang lain. | 0 | 1 | 2 | 3 | 4 |

| | **Masalah/ Kesulitan yang berhubungan dengan : Keuangan Keluarga** | **Tidak pernah** | **Hampir tidak pernah** | **Kadang-kadang** | **Sering** | **Hampir Selalu** |
|---|---|---|---|---|---|---|
| 1 | Sulit untuk keluarga kami merencanakan kegiatan-kegiatan seperti liburan | 0 | 1 | 2 | 3 | 4 |
| 2 | Sulit untuk keluarga kami mendapatkan istirahat yang cukup | 0 | 1 | 2 | 3 | 4 |
| 3 | Menurut saya, keluarga kami mempunyai masalah dengan keuangan | 0 | 1 | 2 | 3 | 4 |
| 4 | Menurut saya,  keluarga kami memiliki banyak masalah | 0 | 1 | 2 | 3 | 4 |
| 5 | Anak saya tidak memiliki peralatan/ alat bantu yang ia butuhkan | 0 | 1 | 2 | 3 | 4 |


Tidak untuk diperbanyak tanpa ijin
Copyright © 1998 JW Varni, Ph.D.
All rights reserved

Tidak untuk diperbanyak tanpa ijin
Copyright © 1998 JW Varni, Ph.D.
All rights reserved

ID# ___________________________

Date:___________________________

# PedsQL ™

## Modul Neuromuskular

Versi 3.0

**LAPORAN REMAJA (Usia 13-18)**

### PETUNJUK

Remaja dengan gangguan neuromuskuler seusia kamu terkadang mengalami masalah yang unik dalam kegiatan sehari-hari. Tolong ceritakan kepada kami **seberapa sering** kamu mengalami **masalah/kesulitan** tersebut dalam **SATU bulan terakhir** ini dengan melingkari salah satu angka berikut untuk setiap masalah/kesulitan yang disampaikan:

**0** Jika **tidak pernah** kamu alami
**1** Jika **hampir tidak pernah** kamu alami
**2** Jika **kadang-kadang** kamu alami
**3** Jika **sering** kamu alami
**4** Jika **hampir selalu** kamu alami

Tidak ada pilihan jawaban yang benar atau salah.
Jika ada pertanyaan yang kamu tidak mengerti, silakan minta bantuan


Tidak untuk diperbanyak tanpa ijin
Copyright © 1998 JW Varni, Ph.D.
All rights reserved

*Dalam **SATU bulan** terakhir, seberapa sering **Masalah/Kesulitan** di bawah ini kamu alami…*

| **Masalah/ Kesulitan yang berhubungan dengan: Penyakit Neuromuskuler Saya** | | **Tidak pernah** | **Hampir tidak pernah** | **Kadang-kadang** | **Sering** | **Hampir Selalu** |
|---|---|---|---|---|---|---|
| 1 | Sulit bernapas | 0 | 1 | 2 | 3 | 4 |
| 2 | Saya mudah sakit | 0 | 1 | 2 | 3 | 4 |
| 3 | Saya mendapat luka dan / atau kemerahan | 0 | 1 | 2 | 3 | 4 |
| 4 | Saya merasakan nyeri pada kaki saya | 0 | 1 | 2 | 3 | 4 |
| 5 | Saya merasakan kecapean (kelelahan fisik) | 0 | 1 | 2 | 3 | 4 |
| 6 | Saya merasakan kaku pada punggung | 0 | 1 | 2 | 3 | 4 |
| 7 | Saya merasa letih ketika bangun tidur | 0 | 1 | 2 | 3 | 4 |
| 8 | Kedua tangan saya lemah | 0 | 1 | 2 | 3 | 4 |
| 9 | Saya sulit untuk menggunakan kamar mandi | 0 | 1 | 2 | 3 | 4 |
| 10 | Saya sulit untuk menambah atau menurunkan berat badan saat saya menginginkannya | 0 | 1 | 2 | 3 | 4 |
| 11 | Saya sulit menggunakan kedua tangan saya | 0 | 1 | 2 | 3 | 4 |
| 12 | Saya sulit untuk menelan makanan | 0 | 1 | 2 | 3 | 4 |
| 13 | Saya perlu waktu yang lebih lama untuk mandi atau menggunakan pancuran (*shower*) | 0 | 1 | 2 | 3 | 4 |
| 14 | Saya terluka secara tidak sengaja | 0 | 1 | 2 | 3 | 4 |
| 15 | Saya perlu waktu yang lebih lama untuk makan | 0 | 1 | 2 | 3 | 4 |
| 16 | Saya sulit membalikkan badan saat tidur di malam hari | 0 | 1 | 2 | 3 | 4 |
| 17 | Saya sulit bepergian ke berbagai tempat dengan alat bantu saya | 0 | 1 | 2 | 3 | 4 |

| **Masalah/ Kesulitan yang berhubungan dengan : Komunikasi** | | **Tidak pernah** | **Hampir tidak pernah** | **Kadang-kadang** | **Sering** | **Hampir Selalu** |
|---|---|---|---|---|---|---|
| 1 | Sulit untuk saya menyampaikan apa yang saya rasakan kepada dokter dan perawat. | 0 | 1 | 2 | 3 | 4 |
| 2 | Sulit untuk saya bertanya kepada dokter dan perawat. | 0 | 1 | 2 | 3 | 4 |
| 3 | Sulit untuk saya menjelaskan penyakit saya kepada orang lain. | 0 | 1 | 2 | 3 | 4 |

| **Masalah/ Kesulitan yang berhubungan dengan : Keuangan Keluarga Saya** | | **Tidak pernah** | **Hampir tidak pernah** | **Kadang-kadang** | **Sering** | **Hampir Selalu** |
|---|---|---|---|---|---|---|
| 1 | Sulit untuk keluarga saya merencanakan kegiatan-kegiatan seperti liburan | 0 | 1 | 2 | 3 | 4 |
| 2 | Sulit untuk keluarga saya mendapatkan istirahat yang cukup | 0 | 1 | 2 | 3 | 4 |
| 3 | Menurut saya, keluarga saya mempunyai masalah dengan keuangan | 0 | 1 | 2 | 3 | 4 |
| 4 | Menurut saya,  keluarga saya memiliki banyak masalah | 0 | 1 | 2 | 3 | 4 |
| 5 | Saya tidak memiliki peralatan/ alat bantu yang saya butuhkan | 0 | 1 | 2 | 3 | 4 |


Tidak untuk diperbanyak tanpa ijin
Copyright © 1998 JW Varni, Ph.D.
All rights reserved

ID# ___________________________

Date:___________________________

# PedsQL ™

## Modul Neuromuskular

Versi 3.0

**LAPORAN ORANGTUA DENGAN REMAJA** (**Usia 13-18**)

**PETUNJUK**

Remaja usia 13-18 tahun dengan gangguan neuromuskular terkadang mengalami masalah yang unik dalam kegiatan sehari-harinya. Tolong sampaikan pada kami **seberapa sering masalah/ kesulitan** tersebut dialami anak remaja Anda selama **SATU bulan terakhir**, dengan melingkari angka berikut ini untuk setiap masalah/ kesulitan yang dialami:

**0** jika **tidak pernah** dialami
**1** jika **hampir tidak pernah** dialami
**2** jika **kadang-kadang** dialami
**3** jika **sering** dialami
**4** jika **hampir selalu** dialami

Tidak ada jawaban benar atau salah.
Jika Anda tidak mengerti suatu pertanyaan, silahkan minta bantuan.


Tidak untuk diperbanyak tanpa ijin

Copyright © 1998 JW Varni, Ph.D.
All rights reserved

*Dalam **SATU bulan** terakhir, seberapa sering **Masalah/Kesulitan** berikut ini dialami oleh anak Anda …*

| **Masalah/ Kesulitan yang berhubungan dengan: Penyakit Neuromuskuler Anak Saya** | | **Tidak pernah** | **Hampir tidak pernah** | **Kadang-kadang** | **Sering** | **Hampir Selalu** |
|---|---|---|---|---|---|---|
| 1 | Kesulitan bernapas pada anak saya | 0 | 1 | 2 | 3 | 4 |
| 2 | Anak saya mudah sakit | 0 | 1 | 2 | 3 | 4 |
| 3 | Munculnya luka dan/ atau kemerahan pada anak saya | 0 | 1 | 2 | 3 | 4 |
| 4 | Nyeri pada kaki anak saya | 0 | 1 | 2 | 3 | 4 |
| 5 | Kecapean (kelelahan fisik) yang dirasakan anak saya | 0 | 1 | 2 | 3 | 4 |
| 6 | Rasa kaku pada punggung anak saya | 0 | 1 | 2 | 3 | 4 |
| 7 | Anak saya bangun tidur dalam keadaan letih | 0 | 1 | 2 | 3 | 4 |
| 8 | Kelemahan pada kedua tangan anak saya | 0 | 1 | 2 | 3 | 4 |
| 9 | Sulit untuk anak saya menggunakan kamar mandi | 0 | 1 | 2 | 3 | 4 |
| 10 | Sulit untuk anak saya menambah atau menurunkan berat badan saat dia menginginkannya | 0 | 1 | 2 | 3 | 4 |
| 11 | Sulit untuk anak saya menggunakan kedua tangannya | 0 | 1 | 2 | 3 | 4 |
| 12 | Sulit untuk anak saya menelan makanan | 0 | 1 | 2 | 3 | 4 |
| 13 | Anak saya membutuhkan waktu yang lebih lama untuk mandi atau menggunakan pancuran (*shower*) | 0 | 1 | 2 | 3 | 4 |
| 14 | Anak saya terluka secara tidak sengaja | 0 | 1 | 2 | 3 | 4 |
| 15 | Anak saya perlu waktu yang lebih lama untuk makan | 0 | 1 | 2 | 3 | 4 |
| 16 | Sulit untuk anak saya membalikkan badannya saat tidur di malam hari | 0 | 1 | 2 | 3 | 4 |
| 17 | Sulit untuk anak saya bepergian ke berbagai tempat dengan alat bantunya | 0 | 1 | 2 | 3 | 4 |

| **Masalah/ Kesulitan yang berhubungan dengan : Komunikasi** | | **Tidak pernah** | **Hampir tidak pernah** | **Kadang-kadang** | **Sering** | **Hampir Selalu** |
|---|---|---|---|---|---|---|
| 1 | Sulit untuk anak saya menyampaikan apa yang ia rasakan kepada dokter dan perawat. | 0 | 1 | 2 | 3 | 4 |
| 2 | Sulit untuk anak saya bertanya kepada dokter dan perawat. | 0 | 1 | 2 | 3 | 4 |
| 3 | Sulit untuk anak saya menjelaskan penyakitnya kepada orang lain. | 0 | 1 | 2 | 3 | 4 |

| **Masalah/ Kesulitan yang berhubungan dengan : Keuangan Keluarga** | | **Tidak pernah** | **Hampir tidak pernah** | **Kadang-kadang** | **Sering** | **Hampir Selalu** |
|---|---|---|---|---|---|---|
| 1 | Sulit untuk keluarga kami merencanakan kegiatan-kegiatan seperti liburan | 0 | 1 | 2 | 3 | 4 |
| 2 | Sulit untuk keluarga kami mendapatkan istirahat yang cukup | 0 | 1 | 2 | 3 | 4 |
| 3 | Menurut saya, keluarga kami mempunyai masalah dengan keuangan | 0 | 1 | 2 | 3 | 4 |
| 4 | Menurut saya, keluarga kami memiliki banyak masalah | 0 | 1 | 2 | 3 | 4 |
| 5 | Anak saya tidak memiliki peralatan/ alat bantu yang ia butuhkan | 0 | 1 | 2 | 3 | 4 |


Tidak untuk diperbanyak tanpa ijin
Copyright © 1998 JW Varni, Ph.D.
All rights reserved

Tidak untuk diperbanyak tanpa ijin
Copyright © 1998 JW Varni, Ph.D.
All rights reserved

ID# ____________________________

Date:___________________________

# PedsQL ™

## Modul Neuromuskular

Versi 3.0

**LAPORAN DEWASA MUDA (Usia 18-25)**

**PETUNJUK**

Dewasa muda dengan gangguan neuromuskuler seusia kamu terkadang mengalami masalah yang unik dalam kegiatan sehari-hari. Tolong ceritakan kepada kami **seberapa sering** kamu mengalami **masalah/kesulitan** tersebut dalam **SATU bulan terakhir** ini dengan melingkari salah satu angka berikut untuk setiap masalah/kesulitan yang disampaikan:

**0** Jika **tidak pernah** kamu alami
**1** Jika **hampir tidak pernah** kamu alami
**2** Jika **kadang-kadang** kamu alami
**3** Jika **sering** kamu alami
**4** Jika **hampir selalu** kamu alami

Tidak ada pilihan jawaban yang benar atau salah.
Jika ada pertanyaan yang kamu tidak mengerti, silakan minta bantuan


Tidak untuk diperbanyak tanpa ijin
Copyright © 1998 JW Varni, Ph.D.
All rights reserved

*Dalam **SATU bulan** terakhir, seberapa sering **Masalah/Kesulitan** di bawah ini kamu alami…*

| | **Masalah/ Kesulitan yang berhubungan dengan : Penyakit Neuromuskuler Saya** | **Tidak pernah** | **Hampir tidak pernah** | **Kadang-kadang** | **Sering** | **Hampir Selalu** |
|---|---|---|---|---|---|---|
| 1 | Sulit bernapas | 0 | 1 | 2 | 3 | 4 |
| 2 | Saya mudah sakit | 0 | 1 | 2 | 3 | 4 |
| 3 | Saya mendapat luka dan / atau kemerahan | 0 | 1 | 2 | 3 | 4 |
| 4 | Saya merasakan nyeri pada kaki saya | 0 | 1 | 2 | 3 | 4 |
| 5 | Saya merasakan kecapean (kelelahan fisik) | 0 | 1 | 2 | 3 | 4 |
| 6 | Saya merasakan kaku pada punggung | 0 | 1 | 2 | 3 | 4 |
| 7 | Saya merasa letih ketika bangun tidur | 0 | 1 | 2 | 3 | 4 |
| 8 | Kedua tangan saya lemah | 0 | 1 | 2 | 3 | 4 |
| 9 | Saya sulit untuk menggunakan kamar mandi | 0 | 1 | 2 | 3 | 4 |
| 10 | Saya sulit untuk menambah atau menurunkan berat badan saat saya menginginkannya | 0 | 1 | 2 | 3 | 4 |
| 11 | Saya sulit menggunakan kedua tangan saya | 0 | 1 | 2 | 3 | 4 |
| 12 | Saya sulit untuk menelan makanan | 0 | 1 | 2 | 3 | 4 |
| 13 | Saya perlu waktu yang lebih lama untuk mandi atau menggunakan pancuran (*shower*) | 0 | 1 | 2 | 3 | 4 |
| 14 | Saya terluka secara tidak sengaja | 0 | 1 | 2 | 3 | 4 |
| 15 | Saya perlu waktu yang lebih lama untuk makan | 0 | 1 | 2 | 3 | 4 |
| 16 | Saya sulit membalikkan badan saat tidur di malam hari | 0 | 1 | 2 | 3 | 4 |
| 17 | Saya sulit bepergian ke berbagai tempat dengan alat bantu saya | 0 | 1 | 2 | 3 | 4 |

| | **Masalah/ Kesulitan yang berhubungan dengan : Komunikasi** | **Tidak pernah** | **Hampir tidak pernah** | **Kadang-kadang** | **Sering** | **Hampir Selalu** |
|---|---|---|---|---|---|---|
| 1 | Sulit untuk saya menyampaikan apa yang saya rasakan kepada dokter dan perawat. | 0 | 1 | 2 | 3 | 4 |
| 2 | Sulit untuk saya bertanya kepada dokter dan perawat. | 0 | 1 | 2 | 3 | 4 |
| 3 | Sulit untuk saya menjelaskan penyakit saya kepada orang lain. | 0 | 1 | 2 | 3 | 4 |

| | **Masalah/ Kesulitan yang berhubungan dengan : Keuangan Keluarga Saya** | **Tidak pernah** | **Hampir tidak pernah** | **Kadang-kadang** | **Sering** | **Hampir Selalu** |
|---|---|---|---|---|---|---|
| 1 | Sulit untuk keluarga saya merencanakan kegiatan-kegiatan seperti liburan | 0 | 1 | 2 | 3 | 4 |
| 2 | Sulit untuk keluarga saya mendapatkan istirahat yang cukup | 0 | 1 | 2 | 3 | 4 |
| 3 | Menurut saya, keluarga saya mempunyai masalah dengan keuangan | 0 | 1 | 2 | 3 | 4 |
| 4 | Menurut saya,  keluarga saya memiliki banyak masalah | 0 | 1 | 2 | 3 | 4 |
| 5 | Saya tidak memiliki peralatan/ alat bantu yang saya butuhkan | 0 | 1 | 2 | 3 | 4 |


Tidak untuk diperbanyak tanpa ijin
Copyright © 1998 JW Varni, Ph.D.
All rights reserved

ID# ___________________________

Date:___________________________

# PedsQL ™

## Modul Neuromuskular

Versi 3.0

### LAPORAN ORANGTUA DENGAN DEWASA MUDA (Usia 18-25)

**PETUNJUK**

Dewasa muda usia 18-25 tahun dengan gangguan neuromuskular terkadang mengalami masalah yang unik dalam kegiatan sehari-harinya. Tolong sampaikan pada kami **seberapa sering masalah/ kesulitan** tersebut dialami anak Anda selama **SATU bulan terakhir**, dengan melingkari angka berikut ini untuk setiap masalah/ kesulitan yang dialami:

**0** jika **tidak pernah** dialami
**1** jika **hampir tidak pernah** dialami
**2** jika **kadang-kadang** dialami
**3** jika **sering** dialami
**4** jika **hampir selalu** dialami

Tidak ada jawaban benar atau salah.
Jika Anda tidak mengerti suatu pertanyaan, silahkan minta bantuan.


Tidak untuk diperbanyak tanpa ijin
Copyright © 1998 JW Varni, Ph.D.
All rights reserved

*Dalam **SATU bulan** terakhir, seberapa sering **Masalah/Kesulitan** berikut ini dialami oleh anak Anda …*

| | **Masalah/ Kesulitan yang berhubungan dengan: Penyakit Neuromuskuler Anak Saya** | **Tidak pernah** | **Hampir tidak pernah** | **Kadang-kadang** | **Sering** | **Hampir Selalu** |
|---|---|---|---|---|---|---|
| 1 | Kesulitan bernapas pada anak saya | 0 | 1 | 2 | 3 | 4 |
| 2 | Anak saya mudah sakit | 0 | 1 | 2 | 3 | 4 |
| 3 | Munculnya luka dan/ atau kemerahan pada anak saya | 0 | 1 | 2 | 3 | 4 |
| 4 | Nyeri pada kaki anak saya | 0 | 1 | 2 | 3 | 4 |
| 5 | Kecapean (kelelahan fisik) yang dirasakan anak saya | 0 | 1 | 2 | 3 | 4 |
| 6 | Rasa kaku pada punggung anak saya | 0 | 1 | 2 | 3 | 4 |
| 7 | Anak saya bangun tidur dalam keadaan letih | 0 | 1 | 2 | 3 | 4 |
| 8 | Kelemahan pada kedua tangan anak saya | 0 | 1 | 2 | 3 | 4 |
| 9 | Sulit untuk anak saya menggunakan kamar mandi | 0 | 1 | 2 | 3 | 4 |
| 10 | Sulit untuk anak saya menambah atau menurunkan berat badan saat dia menginginkannya | 0 | 1 | 2 | 3 | 4 |
| 11 | Sulit untuk anak saya untuk menggunakan kedua tangannya | 0 | 1 | 2 | 3 | 4 |
| 12 | Sulit untuk anak saya menelan makanan | 0 | 1 | 2 | 3 | 4 |
| 13 | Anak saya membutuhkan waktu yang lebih lama untuk mandi atau menggunakan pancuran (*shower*) | 0 | 1 | 2 | 3 | 4 |
| 14 | Anak saya terluka secara tidak sengaja | 0 | 1 | 2 | 3 | 4 |
| 15 | Anak saya perlu waktu yang lebih lama untuk makan | 0 | 1 | 2 | 3 | 4 |
| 16 | Sulit untuk anak saya membalikkan badannya saat tidur di malam hari | 0 | 1 | 2 | 3 | 4 |
| 17 | Sulit untuk anak saya bepergian ke berbagai tempat dengan alat bantunya | 0 | 1 | 2 | 3 | 4 |

| | **Masalah/ Kesulitan yang berhubungan dengan : Komunikasi** | **Tidak pernah** | **Hampir tidak pernah** | **Kadang-kadang** | **Sering** | **Hampir Selalu** |
|---|---|---|---|---|---|---|
| 1 | Sulit untuk anak saya menyampaikan apa yang ia rasakan kepada dokter dan perawat. | 0 | 1 | 2 | 3 | 4 |
| 2 | Sulit untuk anak saya bertanya kepada dokter dan perawat. | 0 | 1 | 2 | 3 | 4 |
| 3 | Sulit untuk anak saya menjelaskan penyakitnya kepada orang lain. | 0 | 1 | 2 | 3 | 4 |

| | **Masalah/ Kesulitan yang berhubungan dengan : Keuangan Keluarga** | **Tidak pernah** | **Hampir tidak pernah** | **Kadang-kadang** | **Sering** | **Hampir Selalu** |
|---|---|---|---|---|---|---|
| 1 | Sulit untuk keluarga kami merencanakan kegiatan-kegiatan seperti liburan | 0 | 1 | 2 | 3 | 4 |
| 2 | Sulit untuk keluarga kami mendapatkan istirahat yang cukup | 0 | 1 | 2 | 3 | 4 |
| 3 | Menurut saya, keluarga kami mempunyai masalah dengan keuangan | 0 | 1 | 2 | 3 | 4 |
| 4 | Menurut saya,  keluarga kami memiliki banyak masalah | 0 | 1 | 2 | 3 | 4 |
| 5 | Anak saya tidak memiliki peralatan/ alat bantu yang ia butuhkan | 0 | 1 | 2 | 3 | 4 |


Tidak untuk diperbanyak tanpa ijin
Copyright © 1998 JW Varni, Ph.D.
All rights reserved
